# 3D Bioprinting of Microbial-based Living Materials for Advanced Energy and Environmental Applications

**DOI:** 10.1021/cbe.4c00024

**Published:** 2024-06-05

**Authors:** Xingqun Pu, Yuqi Wu, Junqiu Liu, Baiheng Wu

**Affiliations:** † College of Material, Chemistry, and Chemical Engineering, Key Laboratory of Organosilicon Chemistry and Material Technology, Ministry of Education, Hangzhou Normal University, Hangzhou 311121, P. R. China; ‡ State Key Laboratory of Chemical Engineering, College of Chemical and Biological Engineering, Zhejiang University, Hangzhou 310027, P. R. China

**Keywords:** 3D bioprinting, microbial-based living materials, bioinks, bioremediation, bioelectricity, biofuel production

## Abstract

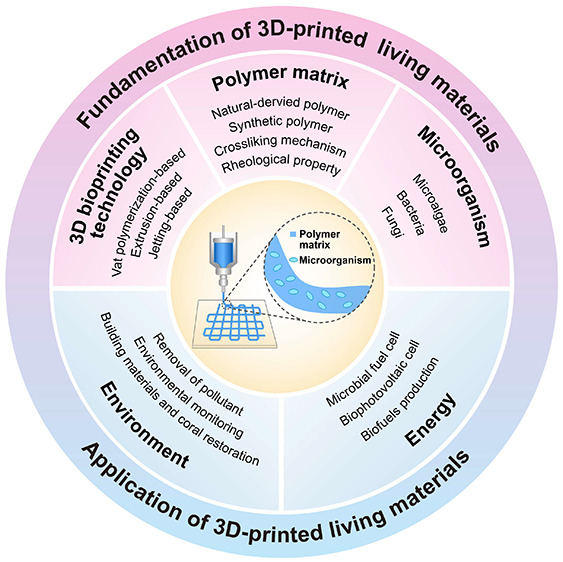

Microorganisms, serving as super biological factories, play a crucial role in the production of desired substances and the remediation of environments. The emergence of 3D bioprinting provides a powerful tool for engineering microorganisms and polymers into living materials with delicate structures, paving the way for expanding functionalities and realizing extraordinary performance. Here, the current advancements in microbial-based 3D-printed living materials are comprehensively discussed from material perspectives, covering various 3D bioprinting techniques, types of microorganisms used, and the key parameters and selection criteria for polymer bioinks. Endeavors on the applications of 3D printed living materials in the fields of energy and environment are then emphasized. Finally, the remaining challenges and future trends in this burgeoning field are highlighted. We hope our perspective will inspire some interesting ideas and accelerate the exploration within this field to reach superior solutions for energy and environment challenges.

## 1 Introduction

As the effort toward sustainable development intensifies, energy and environmental issues have risen to the forefront of global concerns. In recent years, clean energy substitution and environment remediation have emerged as the primary solutions to achieve current carbon reduction targets, thus leading to an increased demand for superior related strategies. Living organisms are natural masters capable of converting energy and producing substances in the most efficient manner. As the oldest inhabitants of earth, microorganisms such as bacteria, fungi, and algae play a crucial role in sustaining higher life forms and foresting the thriving of our planet through energy metabolism and synthesis of essential substances.^1,2^ It can be even asserted that all kinds of complex life activities on earth are fueled by these tiny creatures. Although humans are adept at learning from nature, artificial systems for energy conversion and substances production always fall short in efficiency compared to their natural counterparts. This makes harnessing living cells as factories to address energy and environmental issues a promising area of focus nowadays.

For thousands of years, microorganisms have been exploited by humans for simple synthesis tasks, such as wine brewing or food fermentation.^3^ The rapid development of genetic engineering and synthetic biology has significantly improved the applicability of bioengineering, which enables the use of microorganisms as super factories for producing desired metabolites like pharmaceuticals, valuable chemicals, bioenergy, and providing environmental services such as bioremediation.^2,4,5^ However, microorganisms are sensitive to the stimuli from other cells or external environments and may exhibit self-regulatory behaviors that do not align with human intentions. The challenge of how to regulate living cells to achieve desired functions while maintaining long-term effectiveness and potentially surpassing the efficiency of natural biological processes has always been intriguing to researchers. This necessitates the regulation of metabolism processes at molecular, cell, and bioreactor levels, mitigating adverse effects of external environments and enhancing molecular signal exchange, thus facilitating global optimization and dynamic balance among proliferation, production, and other non-essential cellular processes.

3D bioprinting, as an emerging additive manufacturing technology, has opened new avenues for addressing the aforementioned challenges.^6,7^ The advantage of 3D bioprinting lies in its ability to precisely control of spatiotemporal distribution of microorganisms and chemical components, endowing dynamic characteristics with static functional materials. Besides, it enables site-specific control over the distribution of microorganisms, optimizing intercellular interactions at the population level. By optimizing the structure design of 3D-printed living materials, substrate delivery can be enhanced due to their high surface area and intensified transfer processes, thereby improving biosynthesis efficiency. Therefore, by embedding microorganisms using 3D bioprinting technology, it is possible to create microbial immobilized hydrogel bioreactors with controllable 3D shapes, microstructures, and dynamic metabolic reactions, which offer tremendous potential in sustainable energy production and environmental remediation.

The convergence of 3D bioprinting techniques and microorganisms has opened up unprecedented opportunities for the construction of living materials. Several recent reviews have highlighted the rapid developments in this field and extensively summarized the research progress of living materials with an emphasis on manufacturing methods, synthetic biology tools, spatial confinement regulations, engineered hydrogel and bacterial systems, as well as applications in biomedical area.^1,8−14^ In this review, we start by introducing the latest advancement in 3D bioprinting of living materials powered by microorganisms from a material design perspective, discussing various 3D bioprinting methods, main types of microorganisms used in 3D bioprinting, and key parameters and selection criteria for polymer bioinks. Then we focus on the recent progress made in utilizing such living materials for energy and environmental applications. We are aiming at providing guidance on how to effectively organize different types of cells and inks to achieve desired functionality through 3D bioprinting. We hope our perspective will inspire some interesting ideas and accelerate the exploration within this field to find a superior solution for energy and environment challenges.

## 2 3D Bioprinting Technologies for Living Materials

**Figure 1:**
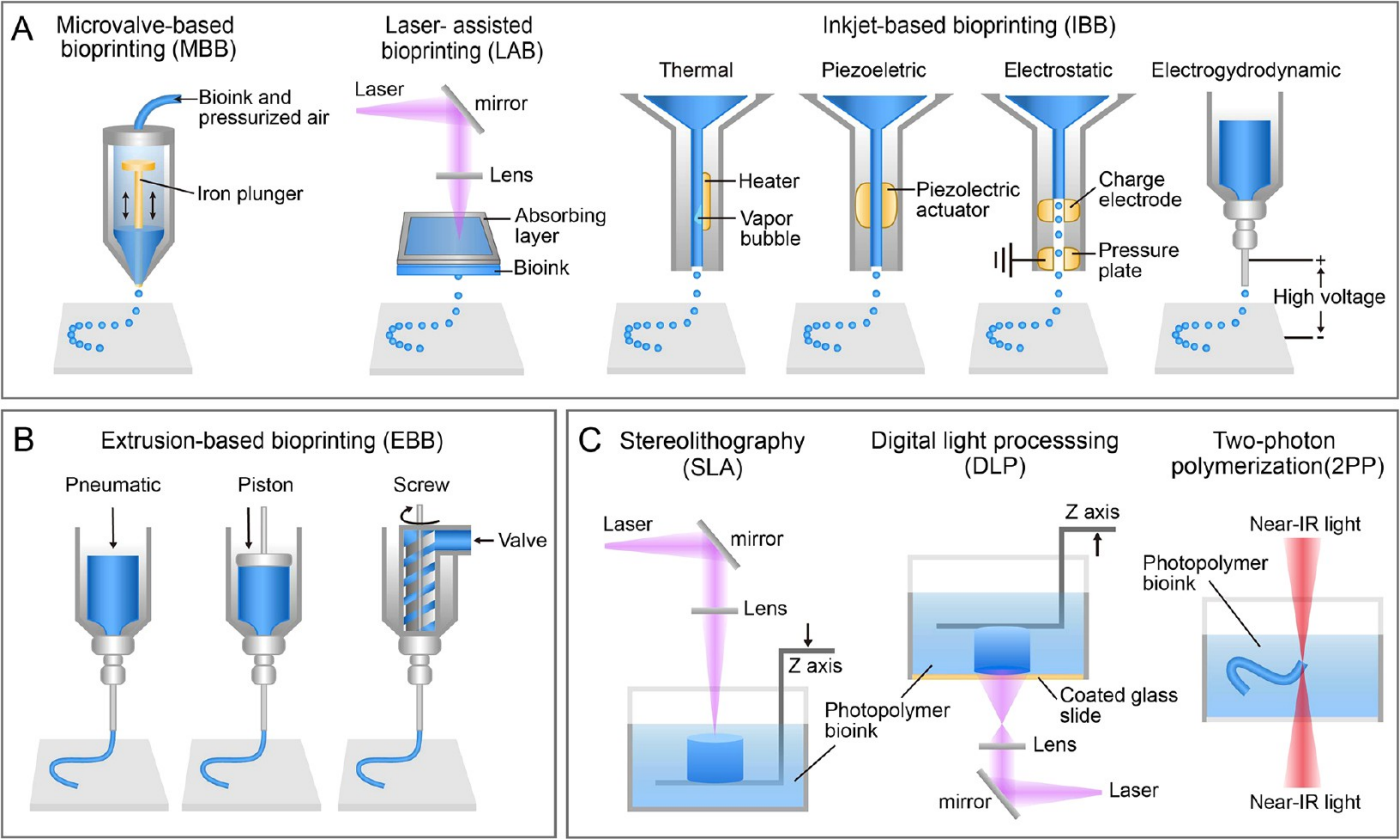
Diagram of three representative 3D bioprinting technologies. (A) Jetting-based bioprinting technology (JBB). (B) Extrusion-based bioprinting technology (EBB). (C) Vat polymerization-based bioprinting technology (VPBB).

3D bioprinting, a technique founded on computer-aided additive manufacturing processes, synergistically combines polymer matrix and living cells as bioinks to create “living materials” with controlled 3D shapes, microstructures, and dynamic metabolic responses. The key element of 3D bioprinting lies in bioinks and the printing technologies used to generate intricate 3D structures. During the printing process, the behavior of cells is influenced not only by the physicochemical properties of the bioink but also by the printing technique employed. Consequently, bioprinting is a technology with numerous influencing factors and complex processes. An ideal 3D printing technology should not only enable the creation of precise geometrical structures but also ensure high cell viability through the printing process. Over the past decade, with the continuous advancement of additive manufacturing technologies, numerous 3D printing techniques suitable for active material fabrication have been developed.^6,15−18^ There are primarily three types of 3D bioprinting techniques: jetting-based bioprinting (JBB),^19,20^ extrusion-based bioprinting (EBB),^21^ and vat polymerization (VP)-based bioprinting(VPBB)^22^ (Figure 1).

### 2.1 Jetting-based 3D Bioprinting

The JBB technology involves the deposition of individual and discrete droplets, encompassing techniques such as microvalve-based bioprinting (MBB), laser-assisted bioprinting (LAB), and inkjet-based bioprinting (IBB).^23^ The MBB system mainly consist of a pressurized reservoir for bioink and a nozzle equipped with electromechanical microvalve controlled holes.^7,24^ The system utilizes pneumatic pressure to overcome the viscosity and surface tension of the fluid, enabling precise deposition of the bioink in the form of droplets. LAB, on the basis of laser-induced forward transfer (LIFT),^36^ works by focusing laser energy onto a specific absorption layer on the glass plate, which subsequently propels the bioink towards the receiving substrate in the form of high-pressure liquid droplets. LAB overcomes the limitations of nozzle-based bioprinting systems due to its nozzle-free features. This enables flexible printing of living materials without being restricted by the concentration and viscosity of bioink. Nevertheless, the high cost and time-consuming nature of the process remain as barriers to its application.^25^

IBB initially derived from commercial inkjet printers, is one of the most commonly used technique within JBB technology.^20^ It is mainly categorized into drop-on-demand and continuous inkjet printing methods. Between them, continuous inkjet printing, which continuously generates droplets regardless of the actual need, results in wastage and potential recycling pollution. Therefore, it is not currently used for living materials printing and thus IBB typically refers to drop-on-demand inkjet bioprinting. In this technology, bioink droplets are generated through mechanical actuation, such as thermal, piezoelectric, electrostatic, or electrohydrodynamic actuation, and precisely deposited onto the predetermined spatial position to form living materials.^19,26,27^ Xu et al. were among the first to adapt IBB for living materials, printing arrays of *E. coli* colonies with specific patterns and density gradients.^28^ The key features of inkjet-based bioprinting are microdropletization, high-throughput, and non-contact nature. It enables the production of small-sized droplets (less than 10 pL), even allowing for single-cell generation, resulting in exceptionally high resolution. In addition, IBB exhibits remarkable printing speed, dispending up to 250 000 droplets per second.^29^ Meanwhile, the print head does not come into physical contact with the printed product or substrate, minimizing the risk of contamination. Despite its numerous advantages, IBB also present some challenges. It is primarily suited for printing bioinks with low viscosity (3–12 mPa/s) and low cell concentration (less than 10^6^ cells/mL).^12^ Consequently, the strength of the printed structures tends to be inferior, and there are challenges in printing vertical structures due to this lower structural integrity. Additionally, during the printing process, microorganisms can be subjected to varying degrees of thermal, mechanical, or shear stress, leading to potential damage. Furthermore, liquid splashing during rapid printing can affect the printing resolution.

In IBB technique, cell viability is influenced by various factors, including cell concentration, bioink viscosity, shear stress, droplet impact velocity, and droplet volume.^30−33^ These factors are not isolated but interconnected and mutually influential. Specifically, cell viability improved with the increasing of cell concentration and bioink viscosity. The improvement is attributed to the fact that a higher cell concentration results in a greater viscous dissipation during droplet formation, which slow down the initial droplet velocity and impact velocity. The low droplet impact velocity reduces the shear forces encountered during droplet propagation, and minimizing cellular deformation caused by impact, thereby, causing a high viability and proliferation capability of printed cells. Moreover, polymer matrix provides an additional buffering effect during the droplet impact on the substrate surface, further decreasing cellular deformation. The cushioning effect is amplified with the increase of bioink viscosity (concentration), contributing to the preservation of cell viability. It is worth mentioning that high cell concentration and bioink viscosity can significantly improve the accuracy of bioprinting by reducing the droplet splashing. However, an increase in bioink viscosity and cell concentration may lead to an elevation in shear stress at the nozzle and during the droplet propagation, resulting in a decrease in cell viability. Therefore, it is imperative to strike a balance among these parameters to achieve optimal printing outcomes. Additionally, the inevitable droplet evaporation during inkjet printing can significantly impact the viability of printed cells. The rate of droplet evaporation is related to the droplet volume and the printing time for each droplet layer. Typically, Smaller droplet volumes and longer printing times per layer lead to shorter drying times for the droplets, resulting in a significant decrease in cell viability. The effect is particularly pronounced when aiming for high printing resolution (small droplet volume). Therefore, maintaining high cell viability while pursuing high printing resolution represents a delicate balancing act that requires careful consideration.

### 2.2 Extrusion-based 3D Bioprinting

The EBB technology has emerged as one of the most promising bioprinting technologies due to its gentle printing conditions, excellent biocompatibility, and broad applicability. EBB operates by extruding bioink in a continuous filament form through a nozzle using pneumatic,^34,35^ piston,^36,37^ or screw systems, which is then accumulated and shaped to a 3D structure. The structure is subsequently solidified through physical or chemical crosslinking.^38^ Unlike IBB technology, EBB can handle a variety of bioinks, including those with high viscosity (up to 10^7^ mPa/s)^39^ or high cell density, making it suitable for fabricating various types of living materials. In addition, EBB enables vertical printing and offers enhanced structural integrity while maintaining good biocompatibility with minimal cellular damage. For instance, Billiet et al. utilized EBB to print gelatin methacrylamide hydrogel embedded with liver cells as bioink, creating artificial liver tissue with a high cell survival rate of up to 97% after printing.^40^ However, the limitations of EBB are also evident. Its printing precision is limited by the physical size of the nozzle, which is typically larger than 100 μm, resulting in a relatively lower resolution unsuitable for printing extremely fine or small structures.^12^

During the EBB process, shear stress is the primary cause of cell damage and death.^41−43^ The cell viability decreases in an astonishing exponential manner as the increase of shear stress. The cells located in close proximity to the nozzle wall experience even stronger shear stress, further exacerbating the degree of cell damage.^44^ Shear stress is related to the factors including printing parameters (nozzle diameter, the geometric shape of nozzle tip, and dispensing pressure) and materials properties (viscosity and rheological properties). By appropriately increasing the nozzle diameter or reducing the dispensing pressure, shear stress can be correspondingly decreased, thereby enhancing cell viability. Under low-pressure conditions, conical nozzles outperform circular nozzles in retaining cell viability.^45^ Additionally, the higher the viscosity of the bioink, the greater the shear stress experienced by the cells, leading to more severe cell damage. Therefore, it is essential for the bioink to possess shear-thinning properties to mitigate excessive fluid shear stress. This not only helps reduce cell damage but also prevent clogging due to high viscosity, ensuring smooth extrusion of cylindrical microfilaments instead of droplets.

### 2.3 Vat Polymerization-based 3D Bioprinting

VPBB involves utilizing lasers (UV, visible, or near infrared light) to selectively crosslink and solidify liquid bioink to construct 3D structures. It is further subdivided into three techniques: stereolithography (SLA),^46,47^ digital light processing (DLP),^48,49^ and two-photon polymerization (2PP).^50−52^ Notably, the printing speed of layer-by-layer photopolymerization in DLP is rapid, compared to point-scanning polymerization in SLA and 2PP, rendering DLP highly suitable for the manufacturing of large-scale living materials.^48^ Due to the exceptional precision of lasers, VPBB achieves a remarkable level of printing accuracy, enabling microscale or even sub-microscale printing resolution (less than 10 μm).^53^ As a nozzle-free bioprinting method, VPBB eliminates shear stress-induced damage to cells while allowing for printing of all cell types. The cell damage mainly comes from light source and the cytotoxicity of photoinitiator. The heat and radiative stress produced by different wavelengths of light sources is harmful to cells, thereby limiting the application of VPBB in microbial bioprinting to a certain extent.^25,54,55^ To achieve stable crosslinking, longer exposure or high light intensity is required, but these conditions can further reduces cell viability.^56,57^ Furthermore, the cytotoxicity of photoinitiators prior to crosslinking can result in severe cell damage, even for the commonly used photoinitiators such as Irgacure 2959 and lithium phenyl-2,4,6-trimethyl-benzoyl phosphinate.^58^ In the field of VPBB, it is crucial to rational select light wavelength, light intensity, crosslinking time, and photoinitiator concentration to ensure the performance of the 3D-printed living materials while minimizing cell damage to the utmost extent.

In summary, each 3D bioprinting technique offers distinct advantages but also faces specific challenges, especially in terms of maintaining the viability and functionality of microorganism cells (Table 1). The choice of printing method mainly depends on the specific requirements of living materials in both of the construction and application processes, covering the type of microorganism cells, polymer inks used, the desired structure, and the intended functionalities. Compare to JBB (microvalve-based bioprinting and IBB) and EBB, the primary advantages of VPBB and LIFT lie in their nozzle-free design and high printing resolution. The nozzle free feature eliminates the risk of shear stress-induce cell damage, ensuring the integrity and viability of cells. However, their major drawback is the high printing costs, which limits their widespread application to some extent. In particular, the selection of suitable bioinks for VPBB is relatively limited, further adding to its limitations in specific applications. In comparison to JBB and VPBB, EBB boasts a significant advantage in printing bioinks with high viscosity and high cell concentration. This characteristic enables EBB to excel in the construction of biological tissues that require high cell concentrations and complex structures. Nevertheless, the disadvantages of EBB include relatively low cell viability (ranging from 75% to 97%) and limited printing precision (20-200 μm), which may compromise the functionality of the final printed structures. When compared to EBB and VPBB, IBB offers the distinct advantage of lower printing costs, making it competitive in cost-sensitive applications. However, the major disadvantage of IBB is its ability to print only low-viscosity (3–12 mPa·s) and low-cell-concentration (<10^6^ cell/mL) bioinks, resulting in weaker structural strength and potentially insufficient for applications that require high mechanical strength. When applying 3D-printed living materials in environmental remediation and energy fields, the requirement for intricate structures is not stringent in most of applications, but the demand for mechanical strength to resist environmental factors is high. At the same time, considering cost factors, EBB has become one of the most widely used bioprinting techniques in environmental and energy applications due to its advantages in printing high-viscosity and high-cell-concentration bioinks, as well as its relatively moderate cost (Table 2).

**Table 1:** Comparison of 3D Bioprinting Technologies^a^

parameters	3D bioprinting technologies			
	JBB		EBB	VPBB
	LAB	MBB and IBB		
bioink viscosity	low (50–150 mPa·s)	low (3–12 mPa·s)	high (30–6 × 10^7^ mPa·s)	medium (1–300 mPa·s)
cell viability	>90%	>85%	40–97%	>95%
cell density	high, >10^8^ cell/mL	low, <10^6^ cell/mL	high, cell spheroids	medium, <10^8^ cell/mL
size of nozzle	nozzle free	2–120 μm	>100 μm	nozzle free
resolution	10–100 μm	10–50 μm	>100 μm	<10 μm
printing process	drop-by-drop	drop-by-drop	line-by-line	layer-by-layer (DLP)
printing speed	fast	fast (1–250 000 droplets/s)	slow (10–50 μm/s)	fast (several layers/s)
printer cost	high	low	medium	high

a Data has been derived from references.^12,16,21,29,59−61^.

**Table 2:** Summary of Common Microorganisms and Polymer Matrix Used in 3D Bioprinting

microorganisms			polymer matrix	
bacteria	microalgae	fungi	natural-derived polymer	synthetic polymer
*E. coli*(47,62−65)	*Coscinodiscus granii*(73)	yeast (*Saccharomyces cerevisiae*^84−88^)	Alginate^37,65,70,72,73,80^	poly(ε-caprolactone) (PCL)^102−104^
*Acetobacter xylinum*(66)	*Cyclotella meneghiniana*(73)	molds	agarose^67^	poly(lactic acid) (PLA)^49,105^
*Bacillus subtilis*(34,46,67,68)	*Fragilaria capucina*(73)	mushrooms	chitosan^65^	polyvinyl alcohol (PVA)^106,107^
*Staphylococcus aureu*	*Chlorella vulgaris*(34,74)		hyaluronic acid (HA)^34,66^	poly(lactic-*co*-glycolic acid) (PLGA)^104,108^
*Photobacterium kishitanii*(69)	*Synechococcus* sp.^75−77^		cellulose^73^	polyethylene glycol (PEG)^109,110^
*Shewanella Oneidensis*(37,70)	*Symbiodinium*(49)		methylcellulose^89^	polyvinylpyrrolidone (PVP)^111,112^
*Pseudomonas putida*(66)	*Marinichlorella kaistiae*(49)		pectin^90^	pluronic F127^87,88,113,114^
*Caulobacter crescentus*,^47^	*Chlamydomonas reinhardtii*(78,79)		carrageenan^91^	gelatin methacrylate (GelMA)^49,115^
*Oceanimonas sp.* XH2^71^	*Synechococcus elongatus*(80)		xanthan^91^	hyaluronic acid methacrylate (HAMA)^49,69^
*Methanobacterium* spp^72^	*Breviolum psygmophilum*(81)		gellan gum^92^	chitosan methacrylate (ChMA)^116^
	*Sporosarcina pasteurii*(36,82)		cyclodextrin^93,94^	hyaluronic acid glycidyl methacrylate (HAGM)^66^
	*Anabaena*(83)		dextran^95,96^	poly(ethylene glycol) diacrylate (PEGDA)^47,49,71,85^
			collagen^97^	Polyethylene glycol dimethacrylate (PEGDMA)^117^
			gelatin^34,68^	
			fibrin^98^	
			silk fibroin^99−101^	

## 3 Bioinks for 3D Printing of Living Materials

In the field of 3D bioprinting, apart from the printing techniques itself, another core challenge lies in discovering bioinks that meet the specific requirements of each application. Bioinks are mainly composed of cells and polymer matrices. The cells contribute functional properties through their physiological activity, while polymer matrix provides a supportive microenvironment for cell growth and subsistence. Table 2 summarizes the common microorganisms and polymer matrix used in 3D bioprinting. Due to the diversity in printing technologies and applications, there is no universal formula for bioinks. Therefore, a balance among printability, cell viability, and functionalities is necessary. In this section, we focus on the commonly used microorganisms and biopolymers in 3D bioprinting, while highlighting the key characteristics that ideal living materials should possess.

### 3.1 Microorganisms Used in Bioink

The functionality of 3D printed living materials is mainly related to the physiological activity of the loaded cells. In this field, microorganisms such as *Escherichia coli* (*E. coli*), and sometimes human cells like human bronchial epithelial cells^118^ or plant cells like *Carrot Calli*(119) typically perform the functions of living materials. Microorganisms, in particular, have garnered significant attention and have been extensive researched within the context of microorganism-based 3D printed living materials, due to their proliferation, low cultivation costs, and ease of use in synthetic biology.^120^

Microorganisms, encompassing a diverse group of organisms such as bacteria, fungi, and algae, play a vital role in various fields including material science, medicine, and food industry. The emergence of biohybrid materials, which integrate biocompatible substances with microorganisms, has become an intriguing research branch at the intersection of material engineering and biological sciences. A crucial aspect in this domain lies in leveraging 3D printing technology to precisely manipulate the spatial and temporal distribution of living cells. In this context, our focus centers on three extensively utilized microorganisms in 3D bioprinting: bacteria, microalgae, and fungi.

#### 3.1.1 Bacteria Used in Bioinks

Due to their excellent adaptability to environments, multifunctional metabolic properties, and robust reproductive abilities, bacteria have extensive applications in the field of 3D bioprinting. This includes the utilization of different engineered strains of *E. coli* commonly employed in synthetic biology, *Acetobacter xylinum* (*A. xylinum*) and *Bacillus subtilis* (*B. subtilis*) used in biomaterials research, as well as pathogens such as *Staphylococcus aureu* (*S. aureus*) employed for antibacterial activity research purposes. The diversity and efficiency exhibited by these bacteria play a significant role in advancing the field of 3D printed living materials.

On the basis of previous research, *E. coli* such as *E. coli* MG 1655,^47,62^*E. coli* BL 21,^65,121,122^ and *E. coli* DH5α,^69,114^ are the most commonly used strains as functional components of bioinks. Besides, *E. coli* MC 1061,^123^*E. coli* JM 109,^124^ and *E. coli* MRR^125^ have also been reported as bioink components. Liu et al. developed living devices that exhibit programmable dynamic functionalities by printing genetically modified *E. coli* into Pluronic F-127 hydrogel matrix.^114^ These living devices include biological logic gates, intricate spatiotemporal response systems, and innovative wearable devices. The engineered *E. coli* strains are programmed to sense signaling chemicals and respond with fluorescence or chemical secretion. For instance, when exposed to signaling chemicals such as *N*-acyl homoserine lactone (AHL), isopropyl β-d-1-thiogalactopyranoside (IPTG), or rhamnose (Rham), certain engineered *E. coli* strains (AHL/GFP+, IPTG/GFP+, or Rham/GFP+) produce green fluorescent protein (GFP), while other strains (AHL/GFP-) repress GFP production. In addition, aTc/AHL+ strain secretes the chemical AHL when detecting anhydrotetracycline. In their approach, they combined various genetically programmed *E. coli* strains with specific chemicals within a meticulously designed 3D hydrogel network, and exploited the interactions between different types of *E. coli* and chemicals in different regions to induce the emergence of complex informational patterns, thereby achieving biological logic gates within living devices. Regarding wearable device applications, they innovated a living tattoo biosensor that leverages the dynamic chemical environment of human skin as an input signal, while the fluorescent response of engineered *E. coli* serves as a visual output signal. This approach opens up new possibilities for real-time, non-invasive monitoring of biochemical signals on the skin surface. Similarly, Macro et al. developed a complex living material with autonomous chemical-sensing capabilities by integrating two distinct bioinks: one containing wild-type bioluminescent bacteria and the other genetically engineered bacteria.^69^ This living material can function as a metabolically powered chemical sensor with an inner hydrogel matrix containing *Photobacterium kishitanii*, emitting blue-green light, and an outer matrix with engineered *E. coli*, featuring by a darkening effect through melanin synthesis. By harnessing the unique metabolic activities of these two types of bacteria, the living material offers a streamlined method for detecting specific chemicals through a simple visual readout. In addition, Duraj et al. have shown that microbial ink, derived exclusively from engineered *E. coli* MG 1655, can be utilized to 3D print various therapeutic living materials, sequestration living materials and regulatable living materials.^62^

In addition to *E. coli*, other bacteria such as *B. subtilis*, *Shewanella oneidensis* MR-1 (*S. oneidensis* MR-1) and *Pseudomonas putida* (*P. putida*) are also used as functional components in 3D printing. González et al. combined agar with *B. subtilis* to develop an antimicrobial 3D printed material capable of surviving in extreme environments without continuous nutrient supply, such as dry conditions and UV exposure.^67^ Freyman et al. utilized 3D bioprinting to develop a hydrogel embedded with *S. oneidensis* MR-1, which can be used as an anode in a microbial fuel cell (MFC).^37^ Schaffner et al. printed living materials containing *P. putida*, capable of degrading phenol into biomass.^66^ In short, almost all bacteria cultured for scientific research can be incorporated into bioinks, significantly expanding the possibilities in the field of 3D bioprinting for living materials.

#### 3.1.2 Microalgae Used in Bioinks

Microalgae are a class of photosynthetic eukaryotic microorganisms that inhabit aquatic environments, including species like diatoms, Chlorella (a type of small green algae), Spirulina, and so on. Some microalgae like Chlorella possess therapeutic properties. Wang et al. printed a living photosynthetic scaffold for use as wound dressing.^126^ This microalgae-laden scaffold, relying on the photosynthetic activity of Chlorella, can continuously produce oxygen under light exposure. When directly applied to chronic diabetic wounds and exposed to light, the scaffold significantly accelerates wound healing by alleviating local hypoxia, and promoting vascular growth and extracellular matrix synthesis. Microalgae are rich in health-beneficial components such as proteins, minerals, vitamins, pigments, fatty acids, sterols, and antioxidants. Uribe-Wandurraga et al. printed Chlorella in cereal, demonstrating the potential of microalgae-enriched 3D printed snacks as functional foods.^127^

#### 3.1.3 Fungi Used in Bioinks

Fungi, which are heterotrophic microorganisms belonging to the eukaryotic group, encompass molds, yeasts, mushrooms, etc. Among these fungi, yeast is particularly prevalent in the field of 3D printing. As a typical facultative anaerobic microorganism, yeast can thrive in both aerobic and anaerobic conditions. It serves as a natural fermenting agent, capable of converting sugars into alcohol and carbon dioxide. These properties of yeast make it significant in the production of self-regenerating materials, alcohol manufacturing, and the food industry. Qian et al. utilized freeze-dried yeast as a primary component in bioinks, supplemented with nanocellulose as a reinforcing filler to create self-supporting porous network structures using 3D printing technology.^85^ This approach achieved efficient cell loading, while effectively facilitating the conversion of glucose into ethanol. The study offers new perspectives on the long-term cultivation of printed biomaterials as biological catalysts. Additionally, Gantenbein et al. have developed a living composite material based on mycelium by 3D bioprinting.^128^ Considering the toughness, flexibility, self-healing ability, and waterproof characteristics of mycelium, this living material shows significant potential for applications in self-repairing robotic skins.

### 3.2 Polymer Matrix Used in Bioinks

Currently, the polymer matrix used for 3D bioprinting mainly includes natural-derived polymers and synthetic polymers. Natural polymers employed in 3D bioprinting are typically sourced from animals, plants, and algal microorganisms. On the basis of their chemical composition, they can be divided into polysaccharides (such as alginate,^37,65,70,72,73,80^ agarose,^67^ chitosan,^65^ hyaluronic acid (HA),^34,66^ cellulose,^73^ methylcellulose,^89^ pectin,^90^ carrageenan,^91^ xanthan,^91^ gellan gum,^92^ cyclodextrin,^93,94^ dextran,^95,96^ etc.) and proteins (such as collagen,^97^ gelatin,^34,68^ fibrin,^98^ silk fibroin,^99−101^ etc.). Synthetic polymers used in 3D bioprinting, originating from the chemical industry, include poly(ε-caprolactone) (PCL),^102−104^ poly(lactic acid) (PLA),^49,105^ poly(vinyl alcohol) (PVA),^106,107^ poly(lactic-*co*-glycolic acid) (PLGA),^104,108^ polyethylene glycol (PEG),^109,110^ polyvinylpyrrolidone (PVP),^111,112^ Pluronic F127,^87,88,113,114^ and so on. The primary advantage of employing natural polymers in 3D bioprinting lies in their inherent bioactivity as well as their high resemblance to the extracellular matrix. These natural polymers exhibit excellent biocompatibility by providing adhesion sites for microorganisms while facilitating their survival and proliferation.^129^ However, the variability among batches of natural polymers often poses challenges in the repeatability of constructing living materials. Additionally, their mechanical properties are generally poor, and even after crosslinking (usually physical crosslinking), the mechanical strength of natural polymers remains limited. In contrast, synthetic polymers can be tailored to specific physical properties, providing better uniformity, repeatability, and mechanical strength, that facilitate the creation of high-fidelity 3D printed structures. However, synthetic polymers often lack active binding sites for cells exhibit poorer biocompatibility, and their degradation products can be cytotoxic, often leading to reduced cell viability. The properties of these polymer matrix have been described and discussed extensively in a number of recent review articles.^130−134^

In summary, both natural and synthetic polymers possess their respective advantages and disadvantages in the field of 3D bioprinting. It is challenging for a single material, whether natural or synthetic, to simultaneously exhibit the necessary physicochemical properties for 3D printing and the suitable biochemical properties for embedding microorganisms, as these attributes are usually mutually exclusive. Compositing different materials with varying properties can partially address this issue. In practical applications, these materials often need to be selected and combined based on the specific requirements, while also considering rational molecular design and chemical reactions to achieve optimal biocompatibility, degradability, printability, and mechanical properties.

### 3.3 Key Characteristics of Polymer Matrix Used in Bioinks

An ideal bioink should be fully compatible with the printing technology to ensure the constructed structure has high shape fidelity and resolution. It also needs to provide a conducive environment for cell growth and functionality, while simultaneously meeting the mechanical properties and stability requirements of the structure. These prerequisites are directly linked to the physicochemical properties of polymer matrix, including rheological parameters, biocompatibility, and crosslinking mechanisms.

#### 3.3.1 Rheological Property

The rheological properties, such as viscosity, yield stress, and shear-thinning behavior, are critical for the printability of bioink.^42,135^ The successful deposition of bioinks heavily relies on their viscosity, which needs to be adjusted according to different printing techniques. For instance, low-viscosity inks are suitable for IBB to fabricate droplet formation; due to the high fluidity of the ink, rapid gelation during the printing process is required. In contrast, EBB demands high-viscosity inks to prevent droplet formation driven by surface tension. Higher viscosity helps delay the structure collapse, allowing for post-printing gelation but also increases shear stress leading to cell damage. Therefore, it is essential to keep the balance between enhancing printability and preserving cell viability. The viscosity of bioinks is related to the molecular weight and concentration of polymers. High polymer concentrations can lead to dense crosslinking networks, which might inhibit cell proliferation and migration.^9,136^ Thus, the polymer matrix with low concentration and high molecular weight is more suitable for high-viscosity bioink. This explains the prevalence of naturally derived high-molecular-weight polymers in this field, which are often unmatched by synthetic biodegradable polymers. Besides, yield stress—the stress required to initiate fluid flow—is also crucial to prevent cell sedimentation in the bioink chamber during printing, ensuring the homogeneity of 3D-printed living materials. Furthermore, one of the most desirable properties of a bioink is its shear-thinning behavior, where viscosity decreases under shear strain, facilitating easier extrusion, reducing shear stress on cells, and maintaining the resolution of the printed product.^137^

#### 3.3.2 Crosslinking Mechanism

**Figure 2:**
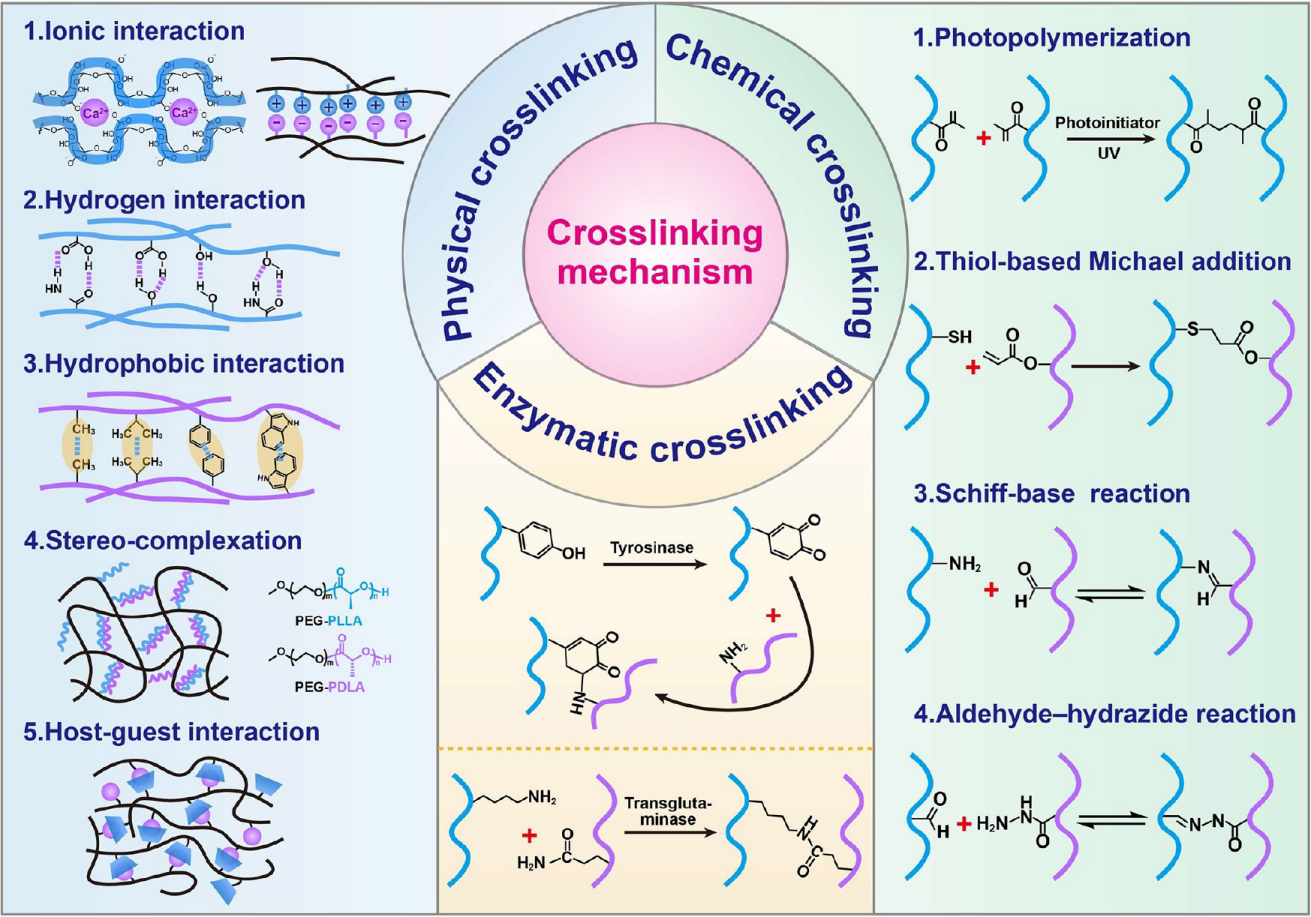
Diagram of crosslinking mechanism of polymer matrix used in bioink.

In various 3D bioprinting technologies, the appropriate crosslinking mechanism of bioinks is one of the key factors for printing living materials with complete structure and functionality. These crosslinking mechanisms mainly involve the crosslinking of bioinks before and after the 3D printing process. The former ensures printability, while the latter imparts mechanical strength and structural stability to the printed structure. The crosslinking method of bioinks is also crucial for preserving the activity and functionality of encapsulated cells. Depending on the mechanism, the crosslinking of polymer matrix can be categorized into physical, chemical, and enzymatic crosslinking (Figure 2).

##### Physical Crosslinking

Physical crosslinking mechanisms rely on non-covalent interactions between polymers, including entanglement, ionic interactions, hydrogen bonding, hydrophobic interactions, stereo-complexation, and host-guest interactions. The initiation of this crosslinking process, typically triggered using physical methods such as mechanical stirring, ultrasonication, or temperature control, results in the transformation of the bioink from a sol state to a gel state. This approach obviates the necessity for potentially hazardous chemical crosslinkers and shows outstanding biocompatibility.

Ionic crosslinking involves electrostatic interactions between polyelectrolytes^74,91,138^ or particles^139^ with opposite charges, as well as metal coordination between polyelectrolytes and multivalent ions.^140^ Anionic polymers include those with negatively charged moieties, such as alginate, kappa (κ)-carrageenan, and xanthan, while cationic polymers contain positively charged moieties, such as chitosan.^91^ For instance, Ng et al. demonstrated the 3D bioprinting of skin tissue constructs by utilizing electrostatic interactions between gelatin and chitosan.^138^ In addition, a prominent illustration of metal coordination involves the application of calcium ions for crosslinking alginate.^64,73^ The gelation process of ionic crosslinking can be precisely controlled and even reversed by changing the bioink pH, which alters the protonation of charged functional groups within the polymer matrix, and by introducing specific chelating agents that effectively remove multivalent ions from the polymer network.

Hydrogen bonding refers to the intermolecular bonding interactions between hydrogen atoms and electronegative atoms, which contribute significantly to the formation of hydrogels in polymer chains. These hydrogels typically have hydrophilic functional groups, including hydroxyl, carboxyl, and amine groups that facilitate hydrogen bonding. On the contrary, when two hydrophobic polymer chains come into proximity, the rearrangement of water molecules induces hydrophobic interactions, resulting in crosslinking to form a hydrogel. Gelation formed through both hydrogen bonding and hydrophobic complexation often exhibits thermo-reversible properties, with their rheological behavior changing in response to temperature variations.^132^ For instance, gelatin-based and agar-based bioinks show disordered, coiled structures at high temperatures but upon cooling they form more ordered conformations aggregating into stable hydrogels.

The stereo-complexation mechanisms are related to stereochemical properties of polymers with opposed chirality. A typical example of this mechanism involves polymer chains comprising repeating units of l- and d-lactic acid.^141−143^ When oligomers of d- and l-lactic acid are coupled with water-soluble polymers, such as dextran^144^ or polyethylene glycol,^145,146^ a stereoselective interlocking system can be formed selectively based on their contrasting chiralities, thereby enhancing hydrogel formation.

Host–guest interaction involves the specific recognition and complexation between host and guest molecules, resulting in the formation of robust and stable complexes.^34,147,148^ A common example is the assembly of cyclodextrins, cyclic oligosaccharides composed of α-1,4-linked d-glucose units, whose hydrophobic internal cavities can accommodate lipophilic guest molecules, contributing to crosslinking.^149^

Physical crosslinking, being dynamically reversible, endows bioinks with unique properties such as reparability, extrudability, and reusability. However, these reversible non-covalent interactions are susceptible to environmental influences, resulting in relatively lower mechanical strength and stability in physical hydrogels.

##### Chemical Crosslinking

Chemical crosslinking primarily relies on the formation of covalent bonds between polymer chains, displaying superior stability and controlled spatiotemporal characteristics in gelation compared to physical crosslinking. The chemical crosslinking mechanisms in bioinks include photo-induced free radical polymerization, thiol-based Michael addition reaction, Schiff’s base reaction, hydrazone-aldehyde coupling, and azide–alkyne cycloaddition, usually triggered by light or heat.

Photo-induced free radical polymerization is a commonly used crosslinking method in 3D bioprinting. This process involves the utilization of addition polymerization reactions that incorporate double-bonded functional groups, often achieved through grafting onto the repeating units or terminal ends of polymer chains. Some of most common (meth)acrylate-modified polymers include gelatin methacrylate (GelMA),^49,115^ alginate methacrylate (AlgMA),^34^ chitosan methacrylate (ChMA),^116^ hyaluronic acid methacrylate (HAMA),^49,69^ hyaluronic acid glycidyl methacrylate (HAGM),^66^ poly(ethylene glycol) diacrylate (PEGDA),^47,49,71,85^ and polyethylene glycol dimethacrylate (PEGDMA).^117^ Under light exposure (visible or UV light), double bonds in these materials react with radicals generated by photoinitiators to form crosslinked networks. By altering the degree of functional group modification, initiator, and monomer concentration, the crosslinking rate and extent can be effectively optimized, thereby improving the physicochemical properties of hydrogel. However, a significant limitation of this route is the potential toxicity of photoinitiators. Currently, only a limited number, including Eosin Y (visible light-initiated) and Irgacure 2959 (UV light-initiated), are FDA-approved.

Thiol-based Michael addition reactions involve nucleophilic additions between thiols and unsaturated functional groups such as acrylates, methacrylates, vinyl sulfones, and maleimides.^150−154^ Under the presence of photoinitiators, thiols can be converted into thiolate anions, which act as radicals initiating chain growth reactions in carbon anions, thereby facilitating the Michael addition process. This gelation method is highly active and selective, with excellent biocompatibility, and has been widely used in engineered living materials. For instance, Skardal et al. used thiolated hyaluronic acid and acrylated four-arm PEG as the main components of bioink. They successfully crosslinked these components via the Michael addition reaction to form shear-thinning hydrogels suitable for 3D bioprinting complex vascular structures.^155^

The Schiff’s base crosslinking mechanism involves the nucleophilic addition reactions between amines and aldehydes to form imine bonds. Amines in natural polysaccharides, amino acids, and other synthetic polymers can undergo dynamically crosslinking with aldehyde-containing small molecules (like glutaraldehyde or genipin) or aldehyde-functionalized polymers under mild conditions. Compared to glutaraldehyde, genipin exhibits superior biocompatibility and has been widely used for crosslinking materials such as gelatin,^156^ collagen,^157^ chitosan,^156,158^ and fibrin.^159^ Du et al. developed a pH-sensitive gelation hydrogel bioink by utilizing Schiff base reactions between gelatin and aldehyde-functionalized dextran.^160^ By regulating the pH level, it becomes possible to control the protonation or deprotonation of amine groups in gelatin, thus fine-tuning the interfacial crosslinking dynamics between gelatin-rich and dextran-rich phases. Consequently, the gelation time can be varied from a few minutes to approximately 1 h.

##### Enzymatic Crosslinking

Enzyme-catalyzed crosslinking is an emerging crosslinking method. Enzymes can catalyze the formation of new reactive groups on polymer chains that may subsequently react with other moieties,^161,162^ or directly catalyze the crosslinking of bioink components.^163,164^ Various enzymes have been used for hydrogel crosslinking, including transglutaminase, horseradish peroxidase, phosphatase alkaline transferase, lysyl oxidase, and tyrosinase.^165^ Da et al. utilized glutaminase transaminase to catalyze the covalent crosslinking between gelatin macromolecule side chains to enhance the stability of 3D printed gelatin/alginate hydrogels.^163^ Enzyme-catalyzed crosslinking reactions occur under mild conditions that can minimize cell death, and the crosslinking process can be directly controlled by adjusting enzyme activity. However, certain types of enzymes, such as transglutaminase and tyrosinase, have limited activity and may result in gels with restricted mechanical strength.

In summary, each crosslinking method offers distinct benefits and limitations, and the choice largely depends on the specific bioprinting method applied. Physical crosslinking methods, generally considered less robust, are predominantly used prior to or during the bioprinting process. In contrast, chemical crosslinking is more commonly employed for the post-processing phase to fix 3D printed constructs. Given that a single crosslinking approach may not meet all practical requirements, integrating multiple crosslinking methods to create a multi-crosslinked gel network can significantly improve the mechanical properties and stability of 3D printed living materials.^71,149,166,167^ The composition of the bioink determines the crosslinking mechanism, implying the need for careful selection of suitable bioinks in 3D printing, developing composite bioinks that integrate various biomaterials, or enhancing the crosslinking sites of biomaterials through physical or chemical methods.

**Figure 3:**
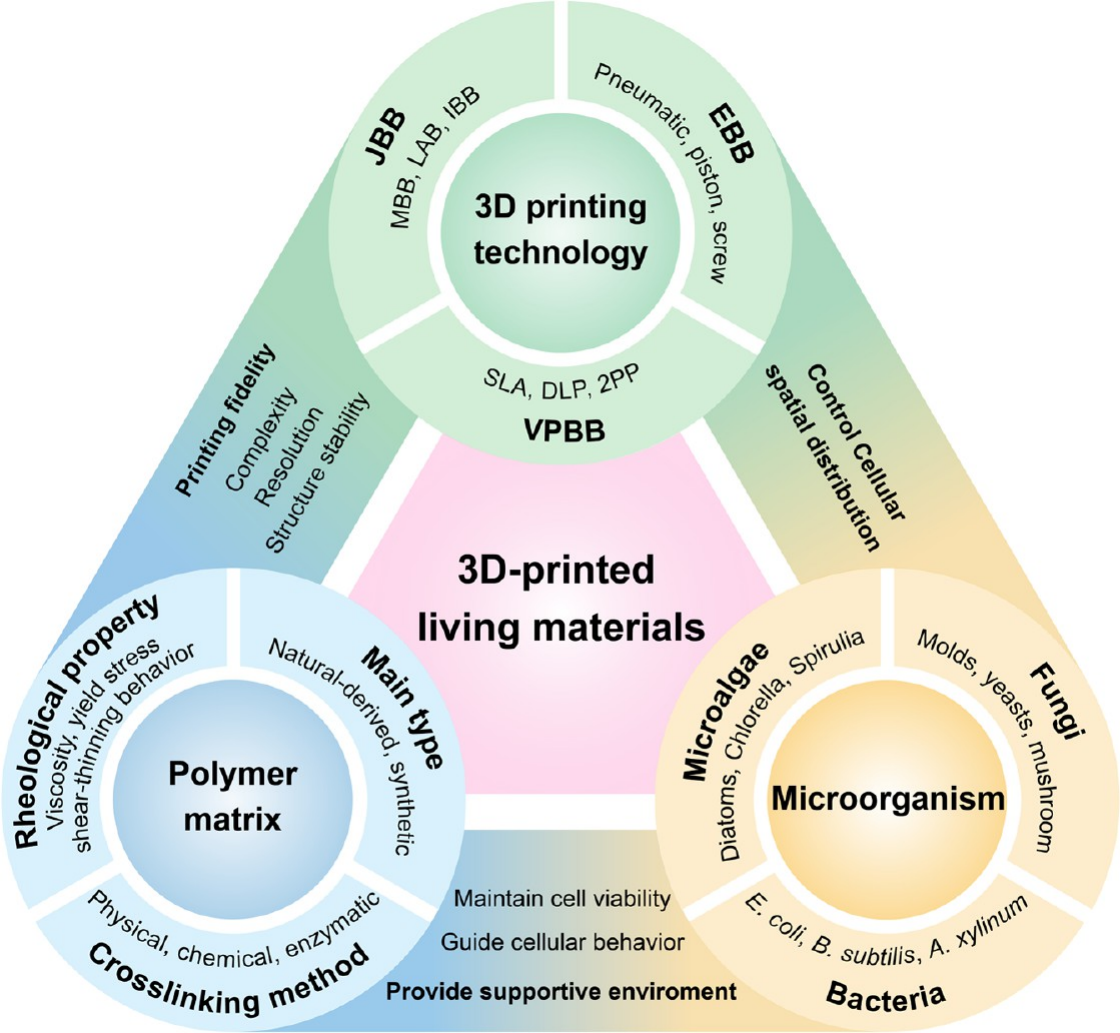
Interdependency of three prime parameters: 3D bioprinting technology, polymer matrix, and microorganism.

The 3D bioprinting technique integrates living cells into polymer matrix to create “living materials”. This process is influenced by the interplay between printing technology, polymer matrix, and the type of cells used (Figure 3). 3D printing affords precise control over the spatial distribution of cells. Polymer matrix provides a suitable environment for cell growth and function, which not only protects the cells from adverse external conditions but also guides cell behavior and facilitates functional performance. Different cell types react differently to the same bioink formulations. Thus, it is essential to choose the proper polymer matrix formulation according to the selected microorganism type based on specific applications. Correspondingly, tuning the rheological property and crosslinking mechanism of the polymer matrix to endow the bioink with excellent printability and biocompatibility, which achieves the fabrication of living materials with complex microstructures and mechanical integrity and stability.

**Figure 4:**
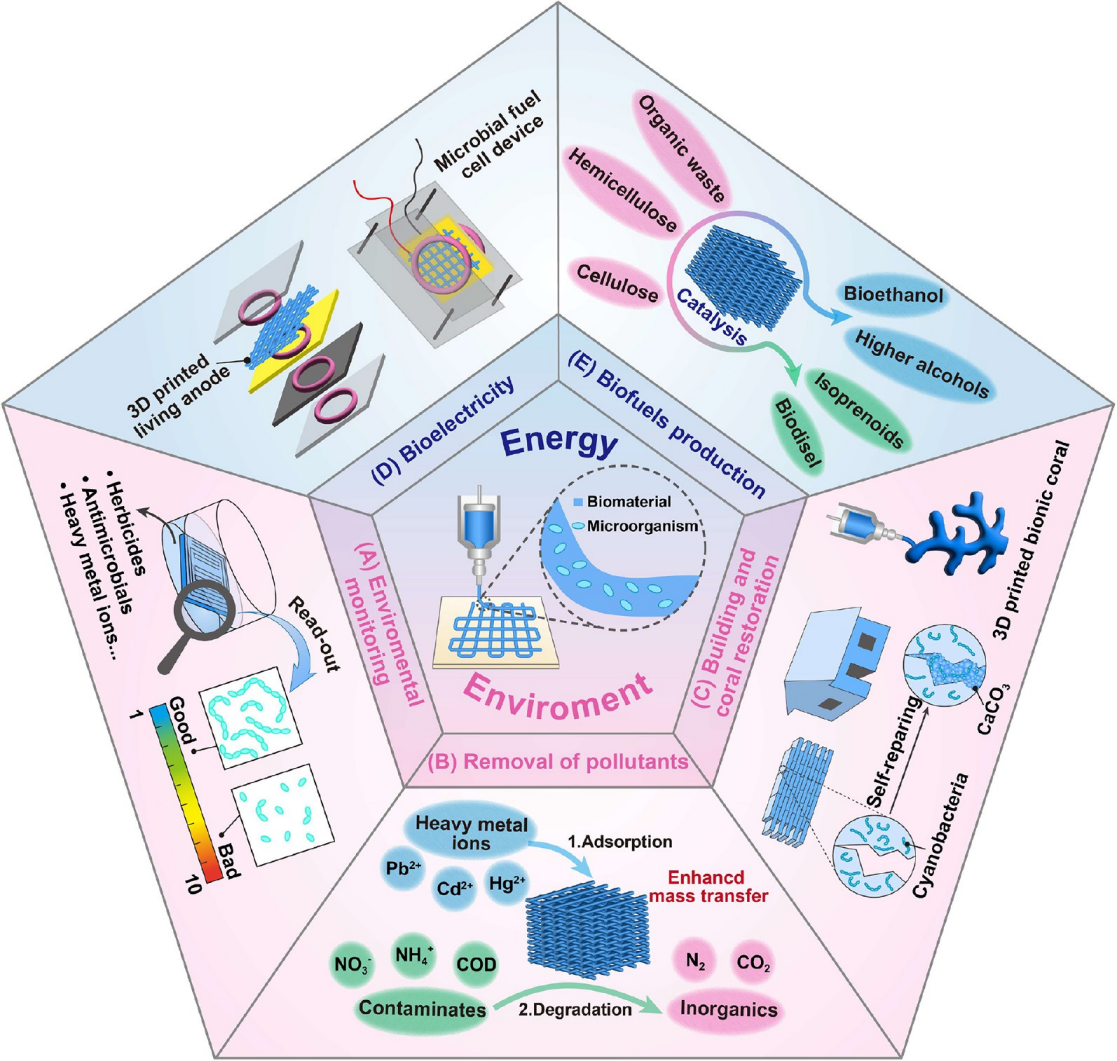
Application of micobial-based 3D-printed living materials in the field of (A-C) environment and (D, E) energy.

The following focuses on the current research on microbial bioprinting in the field of environment and energy (Figure 4). Table 3 summarizes the current research of microbial bioprinting in the field of environment and energy.

**Table 3:** Recent Advances in Microbial-based 3D-Printed Living Materials in the Field of Environment and Energy

3D bioprinting technology	polymer matrix	microorganism	crosslinking mechanism	application	refs
IBB	paper, carbon nanotubes	cyanobacteria (*Synechocystis sp*. PCC 6803)		bioelectricity (BPVs)	(20)
EBB	alginate	cyanobacteria (*S. elongatus* PCC 7942, *Synechocystis sp.* PCC 6803), bacteria (*S. Oneidensis* MR-1, *Methanobacterium* spp)	ion	degradation of organic pollutants, bioelectricity (MFC, BPVs), biofuel production	(80,37,70,72)
	polysiloxane, graphene nanoribbons, PEDOT:PSS	cyanobacteria (*Anabaena*)		bioelectricity (BPVs)	(83)
	alginate, gelation, cellulose nanocrystals	bacteria (*E. coli*)	ion	environmental monitoring	(64)
	alginate, cellulose nanocrystals	diatom (*C. Meneghiniana*, *F. Capucina*)	ion	water quality monitoring	(73)
	alginate, gelation	bacteria (*E. coli*)	ion	degradation of organic pollutants	(65)
	alginate, PVA, PEGDA, nanoclay	bacteria (*Oceanimonas sp*. XH2)	photopolymerization, ion	removal of ammonia	(71)
	HAGA, K-Carrageenan	bacteria (*P. putida*, *A. xylinum*)	photopolymerization	degradation of organic pollutants	(66)
	HAMA, HA with pendant phenylalanine moieties, CB(8)	bacteria (*B. subtilis*)	host–guest, photopolymerization	degradation of organic pollutants	(34)
	alginate, gelation	bacteria (*S. pasteurii*)	ion	manufacturing bionic corals	(36)
	alginate, methylcellulose	cyanobacteria (*Synechococcus sp.* PCC 7002)	ion	manufacturing living building materials	(75)
	PEGDA, nanocellulose	Baker’s yeast (*S. Cerevisiae*)	photopolymerization	bioethanol production	(85)
	hyperbranched PEGDA, thiolated alginate	Baker’s yeast (*S. Cerevisiae*)	Thiol-based Michael addition, ion	bioethanol production	(86)
	pluronic F127 methacrylate	Baker’s yeast (*S.Cerevisiae*)	photopolymerization	bioethanol production	(87,88)
LBB	PEGDA	bacteria (*C. crescentus*)	photopolymerization	rare earth metal recycling, uranium sensing	(47)
	HAGM, GelMA, PEGDA, PLA	microalgae (*Symbiodinium* sp. and *M. kaistiae*)	photopolymerization	manufacturing bionic corals, lipid production	(49)
	Jackfruit aerogel	bacteria (*B. subtilis*)	photopolymerization	degradation of underwater pollutant	(46)

## 4 3D-Printed Living Materials for Environmental Monitoring and Remediation

Nowadays, hazardous pollutants such as toxic gases, heavy metal ions, and organic compounds have been released into the environment, posing a severe threat to the natural environment and human health. Currently, these pollutants are primarily treated through physicochemical techniques like absorption, adsorption, photocatalysis, and filtration.^168^ However, traditional remediation materials often grapple with challenges such as high cost, low efficiency, and the risk of secondary pollution.^120,169^ Consequently, there is an urgent demand for innovative remediation materials with precise detection, low cost, high efficiency, and environmental friendliness. Although microorganisms, including bacteria, algae, and fungi, have demonstrated effectiveness in adsorbing, absorbing, or degrading hazardous pollutants, they are often susceptible to harsh environments.^12,78,170,171^ Fortunately, 3D-printing hydrogel with a grid structure can immobilize microorganisms to protect them from harsh environments. Characterized by their responsiveness, self-repairing ability and programmability, microbial 3D printed living materials have been successfully applied in environmental monitoring and bioremediation.

### 4.1 Environmental Monitoring

**Figure 5:**
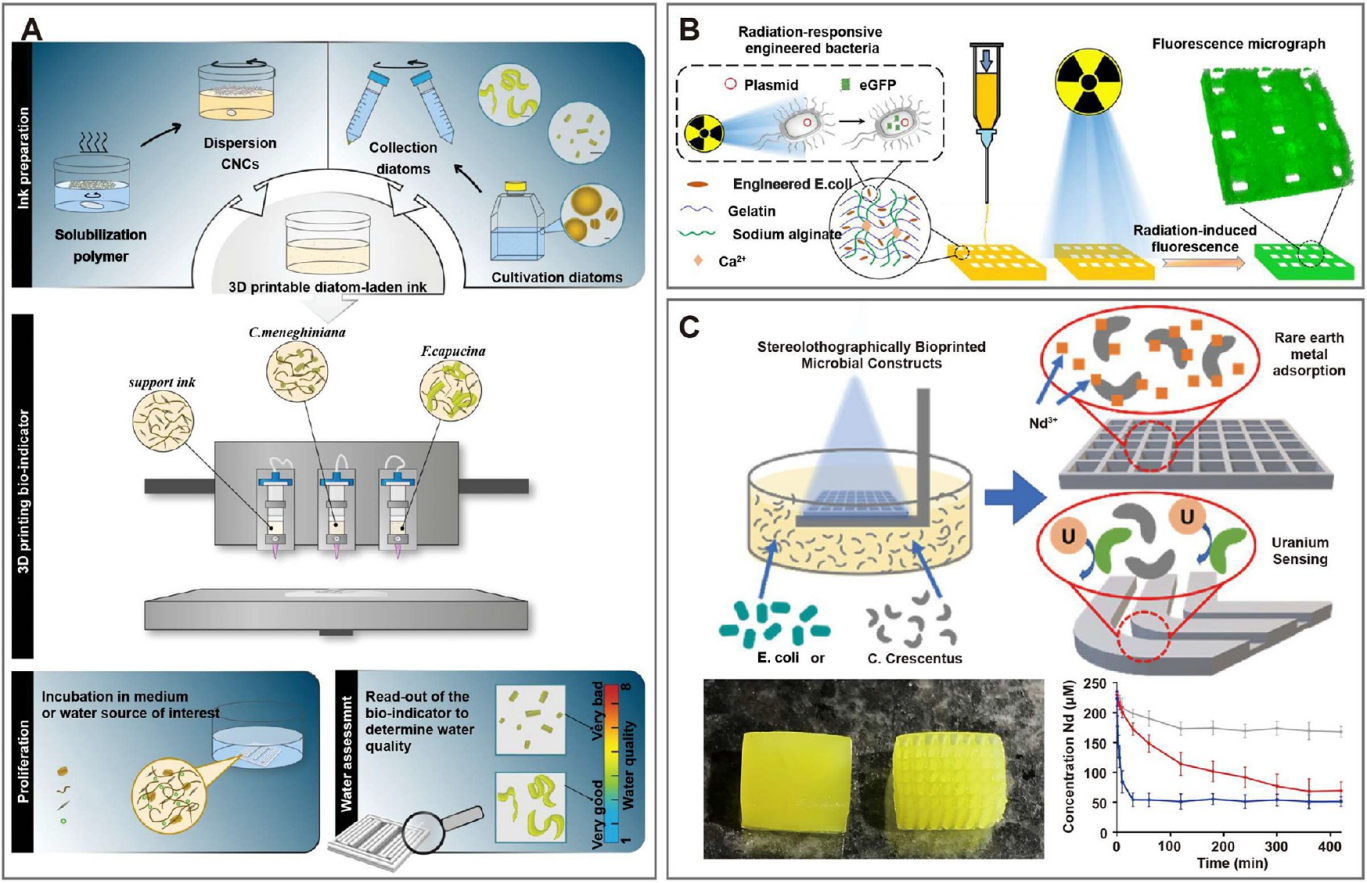
3D-printed living materials for environmental monitoring. (A) The diatom-laden 3D-printed hydrogel for assessment of water contaminants (such as NaCl, herbicide, and anti-microbial agent).^73^ Reproduced with permission from Ref (73). Copyright 2023 WILEY-VCH Verlag GmbH & Co. KGaA. (B) The radiation-responsive 3D-printed hydrogel scaffolds for biological detection of ionizing radiation.^64^ Reproduced with permission from Ref (64). Copyright 2023 Springer Nature. (C) The 3D-printed biofilm as a biosensor for uranium detection.^47^ Reproduced from Ref (47). Available under a CC-BY-NC-ND 4.0 license. Copyright 2021 Dubbin et al.

Microorganism-based 3D printed living materials hold great promise for environmental monitoring, particularly in detecting chemicals, heavy metals, and radioactive substances. Pannier et al. investigated the application of microbial-immobilized living materials for chemical detection.^74^ They incorporated the green microalga *Chlorella vulgaris* (*C. vulgaris*) in alginate solution and applied a non-contact micro-dosage system to print it onto glass substrates, creating patterned cell arrays. Subsequently, these arrays were cross-linked with Ca^2+^ ions or amino-functionalized silica sol. Monitoring the changes in chlorophyll fluorescence monitored through imaging pulse amplitude modulated fluorometry revealed the efficient detection capabilities of hydrogel arrays, particularly for the herbicide atrazine. While this study introduces a novel approach to water quality assessment, the hydrogel array system employed here remains a simple 2D structure and demands specialized equipment. To address this limitation, Boons et al. adopted a bioink consisting of a suspension of diatoms, cellulose nanocrystals, and alginate for 3D printing diatom-laden hydrogels (Figure 5A).^73^ These hydrogel-based living materials serve as a simple and versatile bioindicator for water quality assessment. The study confirmed that the 3D-printed living materials can effectively identify the presence of waterborne contaminants (such as NaCl, herbicides, and antimicrobials) through a simple visual display, demonstrating the potential of this approach as a highly sensitive environmental monitoring technique.

Ionizing radiation is a ubiquitously common phenomenon in daily life and severes as a crucial tool in the medical sector. However, uncontrolled environmental radiation can pose potential risks to ecosystems and human health. Chen et al. developed a living composite hydrogel composed of radiation-responsive engineered bacteria and gelatin/alginate by using 3D bioprinting for biological detection of ionizing radiation (Figure 5B).^64^ The hydrogel carrier not only provides an excellent cultivation environment for engineered bacteria, akin to traditional culture media, but also offers necessary physical support and protection. When exposed to ionizing radiation, the bacteria-laden hydrogel produces green fluorescence, thereby providing real-time reporting of environmental ionizing radiation levels. This innovative approach not only improves radiation detection accuracy but also helps increase public awareness of radiation risks, contributing significantly to scientific research and public health.

Heavy metal pollution is a significant environmental issue due to its association with various pathologies, including cancer, neurodegenerative diseases, and metabolic disorders. Tang at al. propose a biosensor based on genetically modified microorganisms for the detection of heavy metal ions.^63^ The hydrogel-based biosensor has a typical core-shell structure consisting of two parts: an alginate-based hydrogel core that provides a highly hydrated environment for cell growth, and a tough polyacrylamide-based hydrogel shell that protects bacteria against environmental damage. The biosensor, embedded with heavy metal sensing bacteria, the biosensor has been successfully applied to the detection of cadmium ions. Patterning living materials by 3D bioprinting provides the capability to alter and enhance the transport of various components within the structure. Dubbin et al. developed an innovative technique that employs stereolithography for patterning microbes, enabling biofilm printing which allows for printing of biofilm with thickness like natural microbial biofilm (Figure 5C).^47^ The biofilm, embedded with engineered *Caulobacter crescentus* strains, can be used as a biosensor for uranium detection. Despite government limits on uranium, monitoring its levels is still complex. This biosensor offers a cost-effective solution for real-time, on-site uranium measurement in groundwater. Besides, the biofilm exhibits exceptional adsorption and regeneration capability for neodymium with high repeatability and can also be used in rare earth elements recovery.

### 4.2 Removal of Pollutants

**Figure 6:**
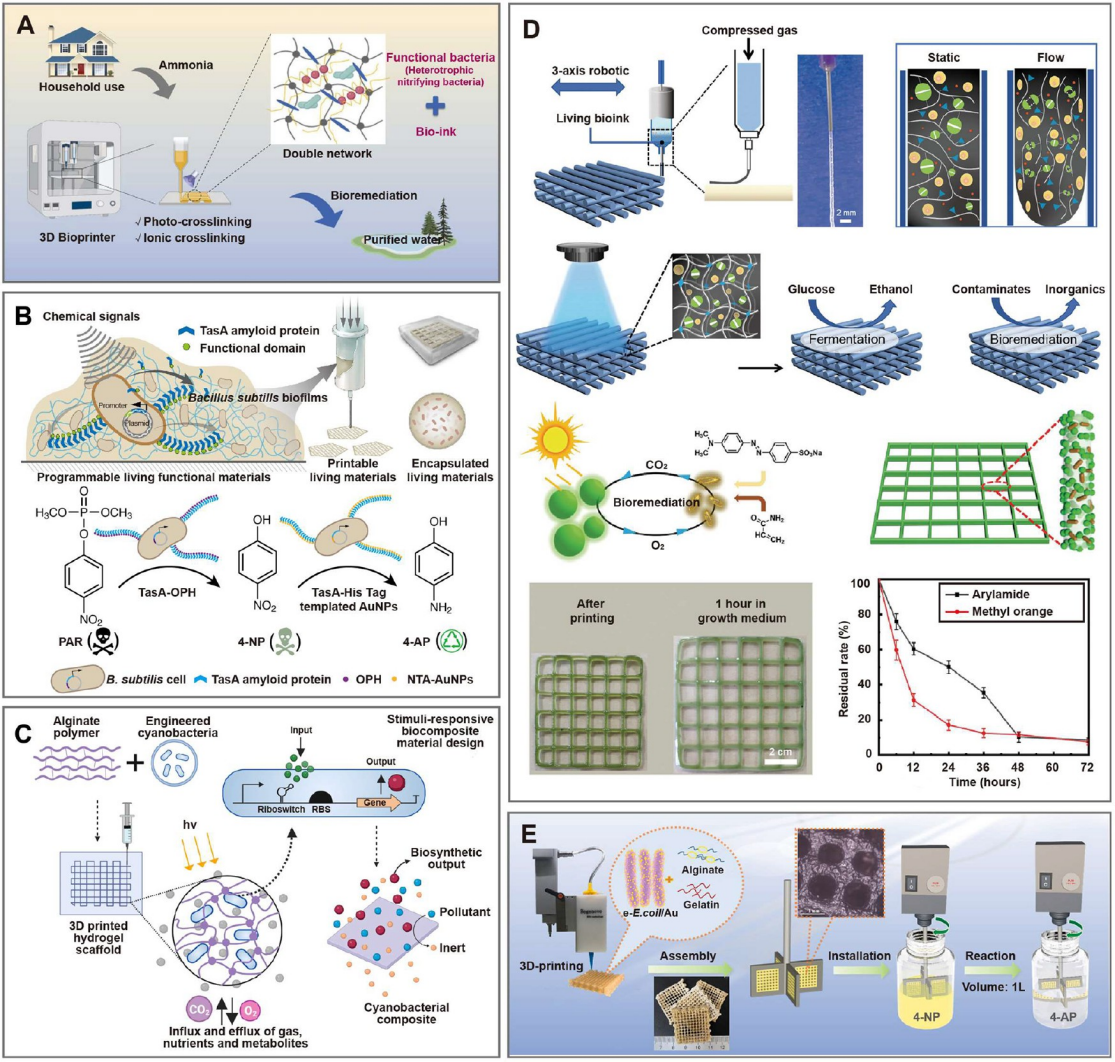
3D-printed living materials for the removal of organic pollutants. (A) The 3D-printed dual-crosslinked scaffolds for the ammonia removal.^71^ Reproduced with permission from Ref (71). Copyright 2022 Elsevier Inc. (B) The 3D-printed engineered bacteria-laden hybrid biofilm for degradation of organic pollutants.^68^ Reproduced with permission from Ref (68). Copyright 2018 Springer Nature. (C) The 3D-printed engineered cyanobacteria-laden hydrogel scaffold for bioremediation.^80^ Reproduced from Ref (80). Available under a CC BY 4.0 license. Copyright 2023 Datta et al. (D) The 3D-printed bacteria-microalgae co-culture system for degradation of organic pollutants.^34^ Reproduced with permission from Ref (34). Copyright 2021 WILEY-VCH Verlag GmbH & Co. KGaA. (E) The 3D-printed living agitating paddle capable of improving mass transfer efficiency for degradation of environmental pollutants.^65^ Reproduced with permission from Ref (65). Copyright 2022 Elsevier Inc.

Microorganisms can efficiently degrade or remove inorganic and organic pollutants through metabolic activities, converting them into relatively harmless substances and facilitating the remediation of soil and water bodies. The utilization of 3D-printed living materials has demonstrated effectiveness in environmental remediation. For instance, Li et al. developed a bioink consisting of a dual cross-linked PEGDA/alginate/PVA/nanoclay composite hydrogel and heterotrophic bacterium (*Oceanimonas* sp. XH2), which was used to print living materials by using IBB technology for the removal of ammonia from wastewater (Figure 6A).^71^ The 3D printed scaffold structure could effectively remove approximately 96% ammonia within 12 h. Impressively, the living material retains the ability to remove ammonia nitrogen after being stored for 168 h under simulated room temperature and culture medium-free conditions. This technology is environmentally friendly, customizable, and reusable, significantly enhancing the application potential of 3D bioprinting in water pollution treatment and other related areas. Schaffner et al. developed a 3D bacterial printing platform that enables additive manufacturing of bacteria-laden hydrogels with arbitrary shapes while precisely controlling the localization and concentration of bacteria.^66^ The 3D printed hydrogel mesh, immobilizing *P. putida*, exhibited an excellent capability of degrading the environmentally harmful phenol into biomass. The living materials can be easily separated from the surrounding medium and reused multiple times, but this strategy compromises the efficiency of phenol degradation. It takes two times of incubations for the phenol degradation rate of living materials to become comparable to that of free-grown liquid culture. In addition, Thakare et al. developed hydrogel filters embedded with algae cells (*Chlamydomonas reinhardtii*) using IBB technology for copper removal from contaminated water.^78^ These algae-laden hydrogel filters achieved an 83% reduction in copper concentration after one hour of filtration. The 3D-printed hydrogel filters hold significant promise for removing other heavy metals like arsenic, cadmium, and lead, through the alteration of the type of algae cells incorporated.

In addition to natural microorganisms such as *P. putida*, genetically engineered bacteria and cyanobacteria are also widely used for the removal of pollutants in environmental bioremediation. Huang at al. leveraged the programmability of *B. subtilis* to engineer a strain capable of producing extracellular TasA-OPH and TasA-HisTag nanofibers, growing into a hybrid biofilm assisted with 3D printing technique (Figure 6B).^68^ By integrating with inorganic gold nanoparticles, such living materials can decompose organophosphorus pesticides into harmless compounds through a two-step biocatalytic cascade reaction. The OPH enzyme catalyzes the pesticide paraoxon (PAR) into intermediate product 4-nitrophenol (4-NP), which is subsequently degraded into harmless 4-aminophenol (4-AP) by HisTag-immobilized gold nanoparticles. This study highlights the great potential of merging genetical engineering and 3D bioprinting in producing advanced biomaterials, showing their promise as an environmentally friendly, efficient, and self-renewing system for environmental remediation. Laccases are multicopper oxidases widely found in organisms like fungi, plants, and insects, known for their ability to decompose azo dyes and various toxic organic pollutants.^172^ Datta et al. used a combination of synthetic biology and 3D bioprinting to develop a stimuli-responsive living material based on genetically engineered cyanobacteria (Figure 6C).^80^ The living material utilizes riboswitch technology to regulate the production of an oxidative laccase enzyme for purifying common textile dye pollutants like indigo carmine. Moreover, the engineered *Synechococcus elongatus* (*S. elongatus*) possesses a “kill switch” mechanism that allows it to self-destruct when its activity is no longer needed, thereby minimizing any potential environmental impact. The development of this programmable living material opens up significant opportunities in the field of environmental bioremediation.

In nature, microorganisms do not work in isolation but instead interact with each other through processes, such as substance metabolism, signal transduction, and gene exchange, thereby forming complex microbial communities.^11,84^ Within these microbial communities, diverse members maintain interdependent and mutualistic relationships. The microbial co-culture system composed of two or more microorganisms can facilitate the synergistic effect among them, and have great potential for improving the degradation efficiency of organic pollutants. For instance, He et al. constructed a bacteria-algae coculture system by using 3D bioprinting technology for bioremediation of chemical substances (Figure 6D).^34^*C. vulgaris* and *B. subtilis* were immobilized in distinguishable hydrogels with dual-network. In this system, *B. subtilis* converts organic chemicals into carbon dioxide while *C. vulgaris* captures carbon dioxide and converts it into oxygen through photosynthesis. The produced oxygen is then utilized by *B. subtilis* for aerobic respiration, creating a mutually beneficial ecological coculture microbial system. Taking advantage of the symbiotic relationship between two different microorganisms, the system demonstrated remarkable capability in degrading acrylamide and methyl orange, showcasing its immense promise for bioremediation.

Although the grid architecture of 3D printing provides a high surface area to maximize contact between bacteria and substrates in the liquid medium, the mass transfer efficiency is not optimal. To further improve the efficiency of mass transfer, Long et al. utilized 3D printing technology to construct a novel catalytic impeller (Figure 6E).^65^ Specifically, they extruded a mixed bioink containing engineered *E. coli*/Au composite and biocompatible polymers (alginate and gelatin) using the direct ink writing method, followed by ion crosslinking to form a robust three-dimensional grid with square symmetry. The monolithic biocatalysts were then assembled into four powered agitating paddles, capable of enhancing mass transfer efficiency and inducing turbulence in the solution. This resulting configuration facilitates the effective reduction of 4-NP in the aqueous phase, a common environmental pollutant, into 4-AP. Furthermore, Yu et al. have developed self-propelling 3D-printed microrobots for the efficient in situ degradation of underwater pollutants, including petroleum-oil, sulfur, nitrogen, and chlorine.^46^ The microrobots are capable of self-propulsion by carbon dioxide (CO_2_) bubbles produced by the reaction of butane-1,4-diol and sodium bicarbonate in water, which enhances the mass transfer efficiency. When approaching the vicinity of the pollutants, the microrobots stop and automatically release bacteria (*B. subtilis*) for precise and efficient bioremediation.

### 4.3 Building Materials and Coral Restoration

In nature, organisms absorb inorganic substances and deposit them as minerals either internally or externally through biomineralization. These minerals, such as calcium carbonate (CaCO_3_) and hydroxyapatite, play a crucial role in organic-inorganic composites like shells, coral reefs, teeth, and bones. Inspired by biomineralization, researchers have harnessed microbially induced CaCO_3_ precipitation (MICP) to construct hybrid living materials. The living materials possess both high mechanical strength and dynamic biological features, showing significant potential in applications such as manufacturing self-healing concrete, eco-friendly building bricks, and biomimetic corals.

**Figure 7:**
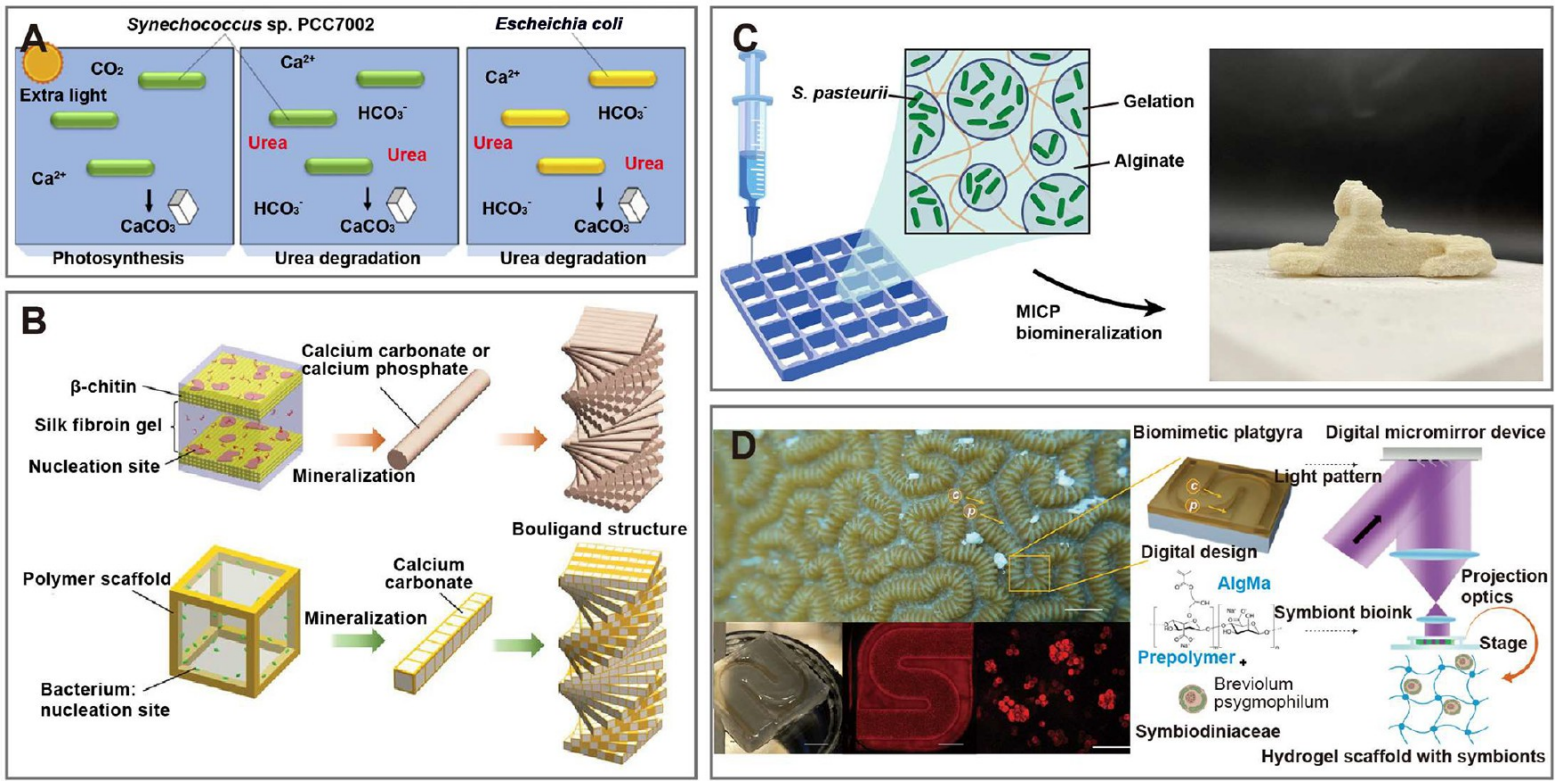
3D-printed living materials for living building materials and coral restoration. (A) Diagram of microbial-induced calcium carbonate precipitation.^76^ Reproduced with permission from Ref (76). Copyright 2021 Elsevier Inc. (B) Bacterial-assisted mineral growth in 3D-printed scaffolds is employed for living building materials.^82^ Reproduced with permission from Ref (82). Copyright 2021 WILEY-VCH Verlag GmbH & Co. KGaA. (C) The 3D-printed biomineral composite for coral restoration.^36^ Reproduced from Ref (36). Available under a CC-BY 4.0 license. Copyright 2023 Hirsch et al. (D) The 3D-printed biomimetic coral.^81^ Reproduced with permission from Ref (81). Copyright 2022 WILEY-VCH Verlag GmbH & Co. KGaA.

Concrete is the most widely used building material in modern construction. However, the production of cement, a key component of concrete, results in significant carbon dioxide emissions, accounting for approximately 8% of global anthropogenic emissions.^173−175^ Recently, research has been focused on developing low-carbon building materials by leveraging microbial-induced calcium carbonate precipitation. For instance, bacteria like *Bacillus pasteurii* (*B. pasteurii*) can secrete urease to catalyze the decomposition of urea, increasing environmental pH and causing calcium carbonate to precipitate around the bacteria (Figure 7A).^76,176−178^ This living material can serve as a cement substitute in concrete. Similarly, cyanobacteria, such as *Synechococcus* sp. strain PCC 7002 and PCC8806, can absorb bicarbonates and carbon dioxide from their surroundings for photosynthesis, leading to an elevation in pH and subsequent precipitation of calcium carbonate (Figure 7A).^179,180^ Heveran et al. developed self-replicating “living” bricks composed of *Synechococcus* sp. PCC 7002, gelatin, and sand, utilizing the CaCO_3_ mineralization ability of photosynthetic cyanobacteria.^181^ The living materials demonstrated excellent mechanical strength, approaching the minimum acceptable strength of Portland cement-based mortars (3.5 MPa). Additionally, Delesky et al. developed an innovative self-healing living building material using a physically crosslinked sand-hydrogel scaffold embedded with two microorganisms—*Synechococcus* and *B. pasteurii*—possessing different biomineralization pathways.^77^ This approach significantly reduces carbon emissions. Despite recent advancements in utilizing microorganisms to enhance traditional materials through biomimetic mineralization, producing mineralizing composite materials with hierarchical structure and life attributes remains challenging. Xin et al. proposed a strategy for creating biomimetic composites by applying bacterial-assisted mineralization in 3D-printed polymer scaffolds (Figure 7B).^82^ In this method, the growth of minerals, induced by urease-producing bacteria in 3D-printed polymer scaffolds, is guided by the microporous structure within the scaffold, ultimately leading to the formation of biomimetic composite materials with pre-designed microstructures. Reinhardt et al. combined 3D bioprinting with cyanobacteria-driven biomineralization.^75^ They integrated *Synechococcus* sp. PCC 7002 into a bioink containing up to 50 wt % sand along with alginate and hydroxyethyl cellulose, which was then printed utilizing an extrusion-based 3D bioprinter. These scaffolds, hosting cyanobacteria, initiate a biomineralization process that results in the creation of living architectural materials with superior mechanical properties. This approach, merging 3D bioprinting with microorganism-induced biomineralization, opens up new avenues for designing and constructing the next generation of eco-friendly building materials.

Coral is a crucial component of marine ecosystems, playing a significant role in maintaining marine biodiversity. However, coral reefs have been severely impacted by climate change and human activities over the past few decades. Some predictions indicate that 60% of global coral reefs will disappear by 2030.^182^ Researchers are actively seeking effective methods to restore coral reefs or create bioengineered alternatives to mitigate the decline. Hirsh et al. used bacteria-loaded microgels as a bioink to fabricate living materials through microbially-induced calcium carbonate precipitation (Figure 7C).^36^ Such biomineral composites can serve as artificial corals to aid in the generation of marine reefs. In addition, Wangpraseurt et al. developed a bioprinting platform capable of 3D printing bionic corals made from a bioink consisting of the microalgae (*Symbiodinium* and *M. kaistiae*), GelMA, and cellulose-derived nanocrystals (Figure 7D).^49^ The artificial bionic corals demonstrated the ability to sustain microalgae at high cell densities. Furthermore, they also created bionic coral tissues and skeletons using 3D bioprinting technology to mimetic the unique structural properties and key diffusion-related processes of coral-algal photosymbiosis in nature.^81^ These findings present opportunities for developing coral-inspired biomaterials with potential applications in coral reef conservation and the study of coral-algal relationships.

## 5 3D-Printed Living Materials for Sustainable Energy

Excessive consumption of non-renewable resources like coal, oil, and natural gas has led to serious energy depletion challenges. This has also caused serious environmental issues, including global warming and soil and water pollution, posing major threats to human survival and development. In response to these challenges, it is imperative to develop sustainable, renewable, and waste-to-energy technologies. Various microorganisms have been discovered with the ability to conversion of sunlight and organic carbon substrates to sustainable energy sources (bioelectricity and biofuels) through metabolic activity. For instance, electroactive microorganisms are able to transform chemical energy into electrical energy by decomposing organic matter to produce electrons and transfer them to external electron acceptors;^183−186^ photosynthetic microorganisms can directly convert solar energy into electrical energy,^187,188^ or convert CO_2_ into biofuels by photosynthesis; while genetically engineered microorganisms can catalyze biomass (cellulose, lignin and etc) into various biofuels.^189^ Microbial electrochemical systems and biofuel production systems, leveraging these organisms, demonstrate immense potential in developing sustainable and eco-friendly energy sources, offering a new strategy to alleviate energy shortages and environmental pollution.

### 5.1 3D-Printed Living Materials for Bioelectricity

**Figure 8:**
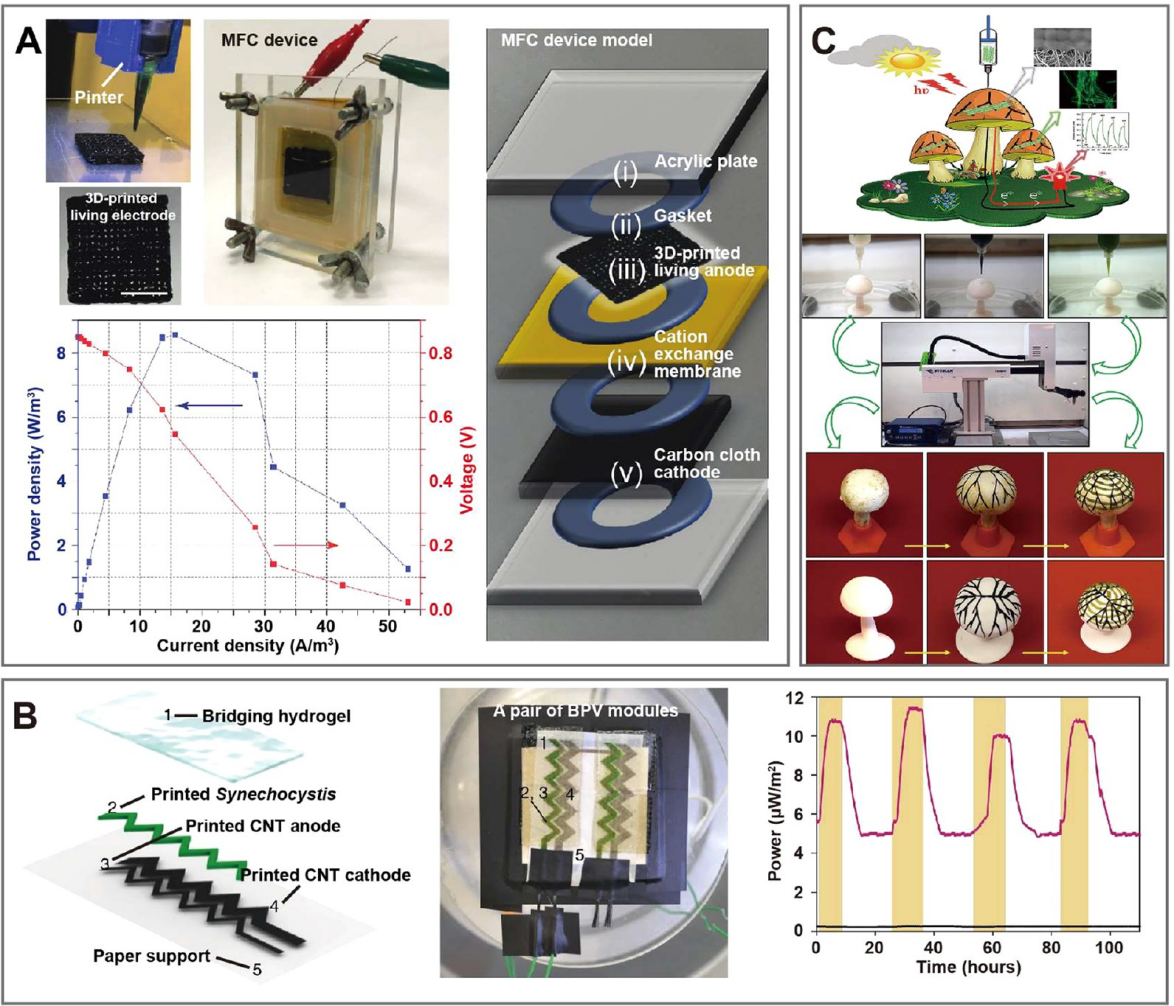
3D-printed living materials for bioelectricity. (A) 3D-printed living electrode for MFC device.^37^ Reproduced with permission from Ref (37). Copyright 2019 Springer Nature. (B) Thin-film paper-based BPV cell containing cyanobacterial cells fabricated by a commercial inkjet printer.^20^ Reproduced from Ref (20). Available under a CC-BY 4.0 license. Copyright 2017 Sawa et al. (C) 3D-printed bionic mushroom embedded with cyanobacterial colonies for photosynthetic bioelectricity generation.^83^ Reproduced with permission from Ref (83). Copyright 2018 American Chemical Society.

Microorganisms typically struggle to grow on unmodified electrodes, limiting the diversity in bioelectrode design. Additionally, precise control over the distribution and quantity of microorganisms on electrodes poses a challenge that impacts the performance of bioelectrodes.^190^ The advancement of 3D bioprinting technology, with its high precision and multifunctionality, allows for the accurate deposition of specific concentrations of microorganisms at targeted locations. This breakthrough opens up novel pathways for developing high-performance microbial electrochemical systems. Freyman et al. incorporated living *S. oneidensis* MR-1 bacteria into conductive ink enriched with carbon black to create a porous bioelectrode by using co-extrusion 3D bioprinting (Figure 8A).^37^ The shape, size, and bacterial concentration of this living anode can be customized through precise control of printing parameters and ink formulation. In MFC, these 3D-printed bioelectrodes serve as anodes, offering twofold benefits. First, the direct integration of conductive pili from electroactive bacteria with the anode enhances electron diffusion, thus facilitating the electron transfer processes of bacteria. Second, the porous network structure of electrodes, characterized by a high surface area, promotes efficient charge transfer between the electrode and the electrolyte. As a result, the MFC device maintains a stable electrical current output for up to 93 h during long-term operation. This research represents a significant leap in microbial electrochemistry, harmoniously integrating microorganisms and electrode materials via advanced 3D bioprinting technology.

Heterotrophic bacteria-based MFC typically rely on the addition of organic carbon sources to support microbial growth. In contrast, biophotovoltaic (BPV) cells, primarily driven by photosynthesis and based on photoautotrophic cyanobacteria or unicellular algae, can convert solar energy into electrical energy using water as the electron source in the absence of organic matter.^187^ Due to their sustainability and eco-friendliness, solar-driven bioelectricity generation strategies are garnering increasing attention. Redox hydrogels encapsulating cyanobacteria have been successfully used for bioelectricity generation.^191^ 3D printing technology aids in the rapid fabrication of these BPV cells. Sawa et al. used a commercial inkjet printer to print cyanobacteria cells onto conductive paper, creating a new type of bioelectrode (Figure 8B).^20^ This electrode can generate power at a rate of 0.38 mW/cm^2^ under light conditions and 0.22 mW/cm^2^ in dark conditions, while maintaining current for over 100 h during light/dark cycles. Simultaneously, it has been demonstrated that this BPV device is capable of continuously powering simple digital clocks and small LED lights. Despite demonstrating significant potential and promise, BPV devices fall short as standalone power sources due to the low electrode transfer efficiency of cyanobacteria cells, resulting in a reduced power density within these BPV systems.^70^ Joshi et al. ingeniously printed cyanobacteria cells and graphene nanoribbons onto mushrooms, creating a biophotovoltaic mushroom for photosynthetic electricity generation (Figure 8C).^83^ The anisotropic and densely packed arrangement facilitated by 3D bioprinting ensured a high cell population density to enable efficient collective action. This precise spatial configuration boosted photocurrent generation, with the 3D-printed cyanobacterial structures achieving an almost eightfold increase in photocurrent compared to isotropic cast cyanobacteria with equivalent seeding densities. Besides, the mushroom provides an ideal habitat for the cyanobacteria cells, while the presence of graphene nanoribbons facilitates extracellular electron transfer.

Leveraging the mutually beneficial symbiotic relationship between photoautotrophic algae and heterotrophic bacteria is anticipated to further improve the electrical performance of BPV devices.^192^ Liu et al. employed 3D printing technology to fabricate a novel hybrid bio-photovoltaic (BPV) device, featuring a dual-layered biofilm of cyanobacteria and heterotrophic bacteria.^70^ In this device, the cyanobacteria produce organic biomass through photosynthesis, which diffuses through the porous structure of the hydrogel to nourish the adjacent layer of heterotrophic bacteria. These heterotrophs then continually generate bioelectricity through respiration. Moreover, the metabolic byproducts of the heterotrophic bacteria, in turn, serve as substrates for the photosynthesis of cyanobacteria, effectively addressing the issue of low electron transfer efficiency in cyanobacteria. The 3D multilayer structure effectively prevents competition among microorganisms while maximizing symbiotic interactions and significantly improving the efficiency of electron transfer among them. This innovative hybrid BPV system, capable of sustainable energy generation without the need for additional organic matter, is ideally suited as a power source for unattended sensors in remote and resource-limited environments.

### 5.2 3D-Printed Living Materials for Biofuels Production

It is worth noting that efficient energy storage is essential for bioelectricity generation.^1^ Another alternative involves the direct production of biofuels through the utilization of microorganisms as cellular factories.^189,193−197^ For instance, Umetsu et al. developed a 3D printed methanogens (*Methanobacterium* spp)-immobilized hydrogel as a living cathode for MCF systems.^72^ Under the supply of CO_2_ and H_2_, the MCF systems showed excellent ability of methane production.

**Figure 9:**
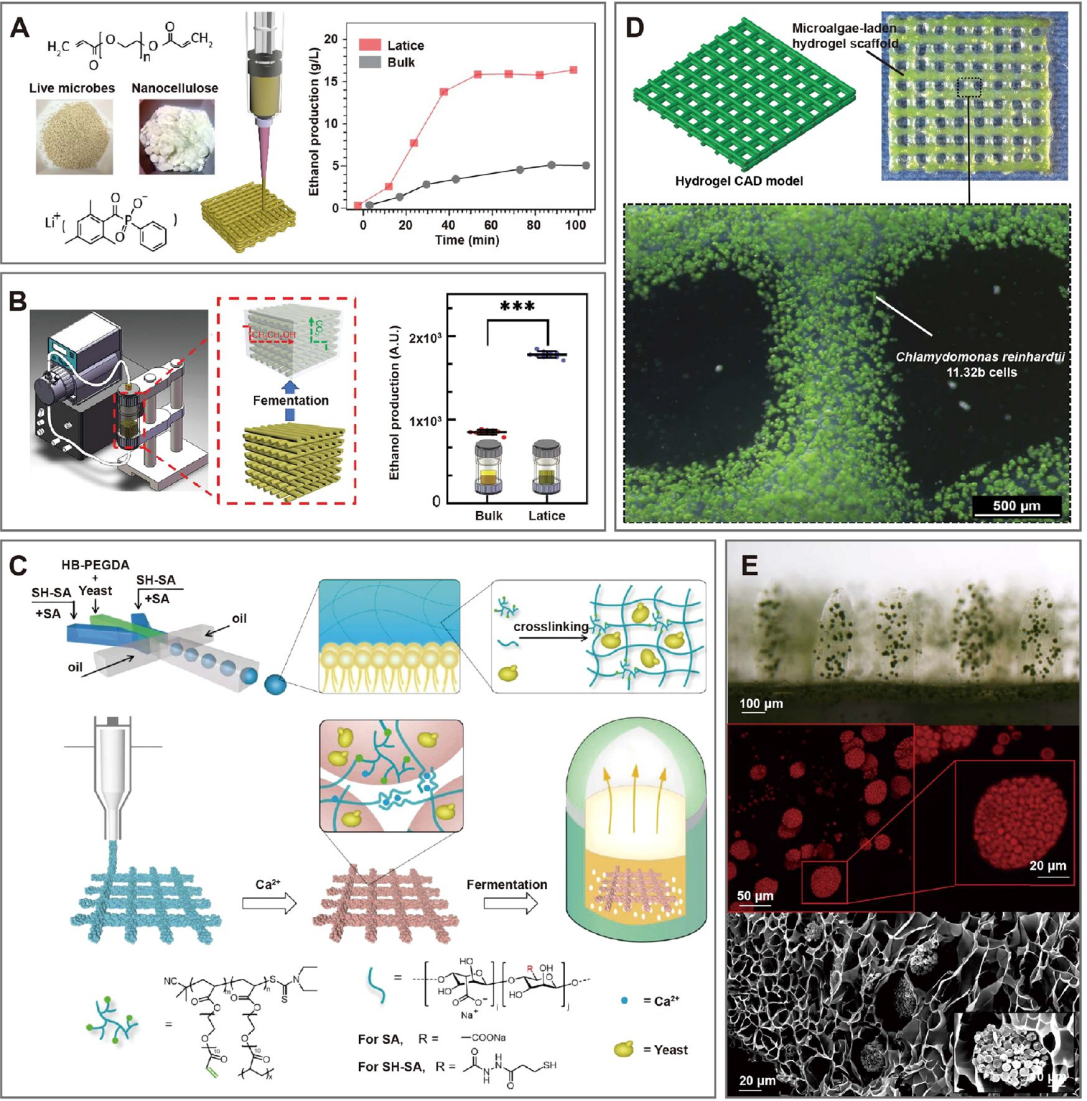
3D-printed living materials for biofuels production. (A) The 3D-printed bio-scaffolds with high yeast density for biocatalytic ethanol production.^85^ Reproduced from Ref (85). Available under ACS Editors’ Choice usage agreement. Copyright 2019 Qian et al. (B) Yeast immobilized dual-crosslinking hydrogel scaffold for ethanol fermentation.^34^ Reproduced with permission from Ref (34). Copyright 2021 WILEY-VCH Verlag GmbH & Co. KGaA. (C) A 3D-printed living material containing yeast-laden microgels for biocatalytic production of ethanol.^86^ Reproduced with permission from Ref (86). Copyright 2022 Elsevier Inc. (D) A 3D-printed hydrogel scaffold designed for the cultivation of microalgae.^79^ Reproduced with permission from Ref (79). Copyright 2015 WILEY-VCH Verlag GmbH & Co. KGaA. (E) A 3D-printed bionic coral for algal cultivation to facilitate the production of biofuels.^49^ Reproduced from Ref (49). Available under a CC-BY 4.0 license. Copyright 2020 Wangpraseurt et al.

Currently, due to the advancement of environmental policies, bioethanol and biodiesel are regaining attention as viable and sustainable alternatives to petroleum-based fuels.^189^ Buteilmann et al. have utilized 3D printing technology to create yeast-laden hydrogels to control the fermentation process, resulting in an increased ethanol yield of 3.7% compared to traditional brewing processes.^87^ Additionally, Qian et al. developed bio-scaffolds with high cell density for biocatalytic ethanol production using 3D printing technology (Figure 9A).^85^ They incorporated freeze-dried yeast as the primary component in the bio-ink, supplemented with nanocellulose as an auxiliary filler. These scaffolds with a porous structure significantly enhanced mass transfer efficiency compared to bulk films, leading to a fourfold increase in ethanol production. Furthermore, He et al. printed double cross-linked hydrogel scaffolds containing yeast, and integrated these bioreactors into a yeast extract peptone dextrose recovery system for maximizing ethanol production rates (Figure 9B).^34^ The emergence of bioprinting technology has indeed spurred the development of living materials with precise design features, however, a challenge remains in maintaining high cell viability while controlling the printability of cell-laden bioinks. Li et al. addressed this by fabricating yeast-laden hydrogel microspheres using droplet microfluidic technology, and then 3D printing them into specifically shaped bio-scaffolds for biocatalytic ethanol production (Figure 9C).^86^ This hydrogel microsphere network effectively encapsulates and compartments the microorganisms, protecting them from shear forces during the 3D printing process and thus ensuring high yeast cell vitality. Compared to bulk hydrogels, these 3D-printed scaffolds carrying Baker’s yeast exhibit an enhanced capacity for ethanol production.

The utilization of photosynthetic microorganisms like microalgae and cyanobacteria holds immense potential in the development of third-generation biofuels.^198,199^ Microalgae, primarily composed of carbohydrates, lipids, and proteins, can be transformed into biofuels through various processes.^200^ Their carbohydrates can be converted into biomethane^201^ or biohydrogen^195,197,202^ through anaerobic digestion, or into bioethanol and higher alcohols like isobutanol,^203^ 1-butanol,^204^ and isopentanol^205^ through fermentation. The lipid content of microalgae, often exceeding 50% of their dry weight,^206^ can be processed into biodiesel, isoprene, and other liquid fuels via transesterification reactions. However, light attenuation caused by the self-shading of algal cells limits photosynthetic efficiency and scalability in algae cultivation for biofuel production.^207^ 3D printing technology has made attractive contributions in overcoming this limitation and enhancing light penetration. Researchers like Krujatz et al. have utilized 3D bioprinting to immobilize algae like Chlorella and Spirulina in hydrogel scaffolds, facilitating their survival and stable growth (Figure 9D).^79^ This innovative technique allows for efficient cultivation of algae even under suboptimal temperature conditions, demonstrating high viability and growth rates.

To optimize light propagation in hydrogels with high algae concentrations, researchers have created biomimetic corals using DLP for cultivating green microalgae drawing inspiration from coral light management.^164,174,175^ This biomimetic coral consists of biocompatible polymer hydrogel (HAGM, GleMA, PEGDA, and PLA) and cellulose nanocrystals (Figure 9E).^49^ With the aid of these nanocrystals and the cup-shaped and cylindrical structures within the coral skeleton, the biomimetic coral outperforms natural coral in light absorption and guiding light toward microalgae. The spatial density of algae in these biomimetic corals can reach up to 10^9^ cells mL^–1^, providing an effective method for large-scale algae production. Furthermore, as mentioned earlier, there exists a mutually beneficial symbiotic relationship between photosynthetic microorganisms and bacteria.^208−210^ Constructing co-cultivation systems based on this relationship enhances photosynthesis and further promotes the growth of microalgae within the hydrogel, thus generating a greater quantity of bioenergy.^211^

Microbial-based 3D-printed living materials exhibit tremendous application potential not only in environmental fields such as pollution monitoring, adsorption, and degradation but also in energy sectors like bioelectricity generation and biofuels production. Particularly noteworthy is their vast possibilities in CO_2_ fixation and conversion. Recently, large-scale microalgae cultivation has been established near power plants, directly sequestering the CO_2_ emitted from these facilities. Leveraging the metabolic characteristics of phototrophic and chemotrophic microorganisms, 3D-printed living materials can mitigate the greenhouse effect and convert CO_2_ in biopolymers (protein, starches, and glucose), biofuels (ethanol and biodiesel), and high-value chemicals (succinic acid, acetone, and isopropyl alcohol). This holds significant importance of achieving “carbon peak” and “carbon neutrality” goals.

For instance, 3D-printed living materials as co-culture systems of bacteria and microalgae can absorb and degrade organic pollutants while effectively sequestering CO_2_.^34,80^ Furthermore, utilizing MICP technology, these 3D-printed living materials can serve as eco-friendly alternative to traditional concrete in construction, while efficiently sequestering CO_2_ from the environment.^75,77^ When applied in biofuel cells and photochemical batteries, 3D-printed materials can effectively utilize CO_2_ and generate biofuel or electrical energy, providing new possibilities for clean energy utilization.^70,187,188,195,196^ The emergence of these living materials not only contribute to the production of clean energy but also offer a novel technological path towards achieving carbon neutrality goals.

Although, research on microbial-based 3D-printed living materials for CO_2_ fixation and conversion is still in its infancy, advancements in genetic engineering and synthetic biology are expected to pave the way for creation of “carbon negative” cell factories capable of capturing and converting it into fuels, chemical products, or building materials with scalable market demand. This will broaden the application prospects of 3D-printed living materials in CO_2_ sequestration and utilization, contributing significantly to the achievement of carbon neutrality goals.

## 6 Conclusion and Future Outlook

In this review, we summarize the design and conception of 3D printing of microbial-based living materials from a material perspective and highlight the latest advancements in their use for environmental and energy applications. The interdisciplinary convergence of polymer chemistry, biochemistry, and material engineering offers opportunities to organize living microorganisms into desired architectures and realize anticipated biological functions. Despite the presence of some incremental achievements, the field of 3D printing of microbial-based living materials is still in its nascent stage, encountering numerous challenges. Further research and development efforts could be directed towards the following aspects.

A comprehensive understanding of the molecular mechanisms underlying biological metabolic pathways in living materials is crucial. The field of environmental and energy applications involves specific tasks related to molecular synthesis or energy conversion through metabolic pathways. Our current understanding of the intricate biosynthetic processes occurring within cells remains incomplete and insufficiently comprehensive. By elucidating the underlying mechanisms, we can significantly enhance targeted interventions through the optimization of substrate components and implementation of artificial catalytic systems, ultimately achieving improvements in both mass production and energy conversion efficiency. Furthermore, by leveraging gene engineering and synthetic biology techniques, the metabolic burden in chassis microorganisms can be reduced and synthesis pathways can be optimized on-demand to augment the production of high-value biochemicals for environmental and energy applications. The emergence of novel bacterial super factories with complex synthetic pathways will also promote the development of multicellular systems by leveraging inherent advantages such as metabolic cooperation, comprehensive substrate utilization, and enhanced tolerance to external environments, showing promising prospects.

Cells are highly sensitive to their external microenvironments, especially in the context of living materials where they are interconnected through polymer matrix. The utilization of 3D printing as a manufacturing tool for fabricating customized structures has shown immense potential in creating an environment conducive to sustaining living microorganisms. However, achieving precise regulation of cellular behavior such as communication, growth, proliferation, and production at the cellular level still remains an insurmountable challenge with current biofabrication methods. This necessitates advancements in 3D bioprinting techniques, particularly in terms of high printing accuracy without compromising versatility for different bioink types or cell adaptability across various geometries and architectures. Apart from technical aspects, structural conception should encompass more innovation rather than solely focusing on providing a high surface area-to-volume ratio. Drawing inspiration from nature by developing biomimetic structures like veins and roots may require multicomponent 3D printing to expand living materials from cellular level to tissue level to organ level—thus leading to a more effective approach for enhancing nutrient and substrate flux while reducing intermediate transmission distance—to ultimately boost biological metabolic efficiency.

The cross-species collaboration among algae, fungi, and bacteria could spark new possibilities in environment and energy applications. Utilizing artificial intelligence to enhance predictions of microbial metabolic interactions and the impact of physicochemical parameters of the polymer matrix (such as spatial constraints and substrate mechanics) on the growth and functionality of microorganisms will aid in constructing microbial co-culture systems, greatly facilitating bioproduction and bioremediation behaviors.

Biofilms formed by microorganisms in nature play a crucial role in monitoring and purifying the environment, but the uneven distribution of nutrients and metabolites limits their functionality. The emergence of 3D bioprinting technology has brought revolutionary breakthroughs to the design and production of biofilms. Compared to traditional preparation methods, the biggest highlight of 3D printing technology lies in its ability to precisely print and arrange different types of bacteria in a controlled three-dimensional space. This gives us the capability to design biofilms with specific shapes, structures, and functions based on actual needs, and accurately realize them through 3D printing technology. Additionally, the standardization, reproducibility, and patterning characteristics of 3D bioprinting technology enable us to achieve mass production of biofilm materials, with each batch meeting uniform quality and performance standards. This significantly enhances the efficiency and stability of biofilm preparation. In the future, we can utilize 3D bioprinting technology to create biofilms with more complex structures and functions, further expanding their application scope in areas such as environmental protection and biomedicine. Simultaneously, through in-depth research on biofilm growth mechanisms and regulatory means, we hope to achieve optimization and improvement of biofilm performance, laying a solid foundation for the widespread promotion of biofilms in engineering applications.

The application of 3D-printed living materials in the environmental field has demonstrated immense potential. However, there are still challenges to overcome in transitioning from the laboratory to practical applications. First, environmental conditions in real-world applications are often more complex and variable compared to controlled laboratory settings. This necessitates that 3D-printed living materials maintain stability and functionality across a range of temperatures, humidity levels, and pH conditions. It requires living materials to be designed and manufactured with the demanding real-world environment in mind, undergoing rigorous testing and validation. Second, production cost remains a significant hurdle for widespread application of 3D-printed living materials. Despite technological advancements that have gradually reduce the cost of 3D printing equipment, high-quality and high-performance environmentally friendly materials often come with a higher price tag. Finding ways to lower material costs without sacrificing performance is crucial for the wider adoption of this technology. Third, efficient mass production and standardization of 3D-printed living materials. Currently, 3D printing technology is still largely limited to small-batch production or customization. Achieving efficient and stable large-scale production, as well as establishing unified production and quality standards, are pressing issues that need to be addressed. Moreover, the field of environmental remediation demands strict long-term performance and safety standards from 3D-printed living materials. This necessitates in-depth research into the biocompatibility, biodegradability, and toxicity of these materials to ensure they do not negatively impact the environment and its ecosystems during practical applications. Ongoing advancements in 3D printing technology, along with the emergence of new materials and processes, are optimizing the performance and cost of 3D-printed living materials. With improved production efficiency and standardization, this technology holds promise for broader applications in the future.

In summary, 3D printing of microbial-based living materials has undoubtedly presented great opportunities in the field of biosynthesis and bioremediation. Future efforts in the research community should strive for interdisciplinary collaboration. We anticipate that the emerging advancements in 3D printing of microbial-based living materials will effectively address environmental concerns and contribute to resolving the energy crisis, ultimately fostering sustainable developments.
